# Validated antimalarial drug target discovery using genome-scale metabolic modeling

**DOI:** 10.1128/aac.00459-25

**Published:** 2025-09-26

**Authors:** Supannee Taweechai, Francis Isidore Garcia Totañes, David Westhead, Clara Herrera-Arozamena, Richard Foster, Glenn A. McConkey

**Affiliations:** 1 School of Biology, Molecular & Cell Biology, University of Leeds 192329, Leeds, United Kingdom; 2 School of Chemistry, University of Leeds 150509, Leeds, United Kingdom; The Children's Hospital of Philadelphia, Philadelphia, Pennsylvania, USA

**Keywords:** malaria, *falciparum*, pyrimidine, CRISPR-Cas, target, druggable, genome-scale metabolic model, flux-balance analysis

## Abstract

Given the rapid resistance of *Plasmodium falciparum* to antimalarial drugs, there is a continual need for new treatments. A genome-scale metabolic (GSM) model was developed with integrated metabolomics and constraint-based, experimental flux-balance data to predict genes essential for *P. falciparum* growth as drug targets. We selected the highly ranked *P. falciparum* UMP-CMP kinase (UCK) to test its necessity and the ability to inhibit parasite growth in the presence of inhibitors. Conditional deletion mutants using the DiCre recombinase system, generated by CRISPR-Cas genome editing, exhibited defective asexual growth and stage-specific developmental arrest. Based on *in silico* and *in vitro* screening, inhibitors were identified that are selective for *P. falciparum* UCK and exhibit antiparasitic activity. This study, for the first time, shows assertions from a GSM model identifying novel, validated “druggable” targets. These findings show a role for GSM models in antimalarial drug discovery and identify *P. falciparum* UCK as a novel, valid malaria drug target.

## INTRODUCTION

Malaria is a significant global health burden, focused on tropical and subtropical regions, with 249 million malaria infections globally and circa 608,000 malaria-related deaths (1). Chemotherapy, the main approach to treatment, is restricted. For example, artemisinin-based combination therapies, currently recommended and widely used for malaria treatment in endemic countries worldwide, exhibited decreased susceptibility in the Greater Mekong subregion in 2009 with delayed parasite clearance (2–5). The rapid and regular arising of resistance necessitates continuous antimalarial drug discovery in conjunction with the identification of novel targets for control of infection.

With the constant need for new antimalarial drugs, methods have been developed focusing on identifying essential genes at the genomic level that may serve as novel targets for drug discovery. A genome-wide saturation mutagenesis method using the *piggyBac* transposon system was applied to *P. falciparum* strain 3D7. The high abundance of TTAA insertion sites in the parasite’s AT-rich genome allows this system to broadly target both coding and noncoding regions (6, 7). Out of 5,399 genes analyzed, 2,680 were found to be essential for asexual blood-stage development. These included known drug resistance-associated genes, such as *K13 Kelch*, *mdr*, and *dhfr-ts*, as well as high-priority drug targets like *pkg* and *cdpk5* (7). Identifying genes whose loss of function results in a severe fitness defect as “essential” in genome-wide knockouts is a first step in drug target validation. Chemical inhibition of parasitic proteins *in vitro* and *in vivo* has been the main driver of novel drug target discovery, such as with PfDHODH (8, 9). Modeling studies have yet to validate this approach by demonstrating asserted genes as essential and can be targeted by inhibitors. A publication of *Plasmodium falciparum* saturation mutagenesis, that was recently extended to *Plasmodium knowlesi* with a considerably higher number of mutations, classified a large collection of genes into essential and nonessential based on subtraction of mutations that do not affect growth (10, 11). This binary classification of genes may generate false-positive essential genes (e.g., due to factors such as growth requirements under different environmental conditions or nonrandom transposon insertions due to chromatin structure), and multiple transposons inserted in a genome may also yield compensatory effects. Hence, directed mutagenesis of single genes is needed for validation. Genome-scale metabolic (GSM) models (also known as GEMs) integrate omics and experimental data to identify targets that are most susceptible to growth inhibition based on flux-balance analysis. Constraint-based models can prioritize enzymes that are most likely to be “druggable” (12). A stage-specific *Plasmodium* GSM model has fostered prioritization of drug targets based on blood stages of infection (13). Their utility in asserting and prioritizing drug targets has been reviewed extensively (12, 14, 15) but has not followed progression of drug target validation from modeling to target validation and druggability. In this study, a constraint-based model of *P. falciparum* was developed to identify potential drug targets. From this list of targets, a top-ranked gene was validated as a drug target. First, a directed, inducible knockout of the gene of interest (GOI) was developed using the DiCre system to test essentiality. The DiCre-recombinase system for conditional gene deletion has become a widely used tool for functional gene analysis in *P. falciparum* (16–26). This system employs Cre recombinase activity to excise *loxP*-flanked DNA sequences in a drug-inducible manner. Initially, the GOI is modified by inserting *loxP* sites at specific positions using CRISPR/Cas9 technology. A DiCre-expressing *P. falciparum* line expresses Cre recombinase as two inactive fragments that dimerize upon drug treatment to form an active enzyme, which excises the targeted DNA segment (21). Subsequently, biochemical assessment of the gene product was performed, and a set of *in silico* predicted inhibitors was screened. Finally, the target was validated by treating parasites with inhibitors and examining parasite growth inhibition.

The research herein investigates the assertion that UMP-CMP kinase (UCK) is a viable drug target for the development of a *P. falciparum* GSM model. The study validates the necessity for *P. falciparum* UCK (PfUCK), investigates its potential as an antimalarial drug target by employing CRISPR-Cas-generated inducible mutants, and assesses its druggability for future drug development. The target validation and rapid screening assay for PfUCK inhibitors will facilitate future high-throughput inhibitor screening.

## MATERIALS AND METHODS

### Metabolic model construction

The three malaria metabolic models that were merged together in this study were those developed by Forth (15), Plata et al. (27), and Huthmacher et al. (14). As the three models utilized different ontological formats for metabolite (i.e., species) and reaction IDs, these IDs were initially converted to SEED format using database files of reaction and species IDs in various ontological formats obtained from metanetx.org (28). The Forth model (iTF143) was used as the base model (also referred to as the minimal model). The Huthmacher et al. (14) and the Plata et al. (27) models served as the source models, i.e., models from which reactions were collected and added into the minimal model. Reactions from the source models must satisfy the following criteria to be added into the minimal model:

1. The reaction has enzyme commission (EC) classification and gene association data.
2. At least one species in the reaction is in the minimal model.

Enzyme commission classification and gene association data were obtained from or verified against the *Plasmodium falciparum* metabolic pathways in Kyoto Encyclopedia of Genes and Genomes database (29) and PlasmoDB (30). To ensure the accuracy of comparing reactions between the three metabolic models, the reaction equations were compared instead of the reaction IDs. Reactions from the source models that satisfy the two criteria were removed from the list of source model reactions and were added into the minimal model. As new reactions were being added to the minimal model, new species from these reactions were also added. Thus, it was possible that reactions that initially satisfied the first criteria, but not the second, could now have a common species in the growing minimal model. The remaining reactions from the source models were repeatedly assessed until no new reaction could be added into the model.

The stoichiometry of the biomass equation was derived using experimentally quantified biomass macromolecular components (DNA, RNA, and proteins) together with published data on the proportions of different subcomponents of these macromolecules. The proportions of individual amino acids were estimated based on published data on the relative abundance of each amino acid in *P. falciparum* (31). Individual deoxynucleotide proportions were estimated using published data on the G/C content of the *P. falciparum* 3D7 genome, while RNA nucleotide proportions were estimated by taking the weighted average of the G/C content of exons and introns (32). The proportions of the carbohydrate subcomponents were adopted from the Forth model (15), while GDP-mannose as part of carbohydrate accumulation was adapted from published *Leishmania major* data (33). GDP-mannose to GDP-fucose ratio was based on data from published experimental quantifications of sugar nucleotides in *P. falciparum* (34). Stoichiometric coefficients (c) for individual reactants involved in the biomass reaction were calculated using the formula (using a single amino acid as an example):


$$c_{aminoacid}=\frac{m_{protein}\times p_{aminoacid}\times {1gDW}_{parasite}\times 10^{3}}{{MW}_{protein}}$$

where

$c_{aminoacid}$= stoichiometric coefficient

$m_{protein}$= gram of protein per gram of parasite dry weight (${gDW}_{parasite}$)

$p_{aminoacid}$= percentage of amino acid in the parasite proteome

${MW}_{protein}$= weighted average molecular weight of parasite protein (gram/mole)

This equation was adopted from that used by Chavali et al. (35) in the development of the genome-scale metabolic model of *Leishmania major*.

### *In vitro* flux measurements

Continuous *P. falciparum* 3D7 *in vitro* cultures were grown in RPMI 1640 growth medium (Life Technologies, UK) supplemented with 5% (wt/vol) Albumax II (Gibco, USA), 0.01% (wt/vol) hypoxanthine (Sigma, USA), and 0.1% (vol/vol) gentamicin at 5% hematocrit (O + blood obtained from the National Blood Service of the NHS Blood and Transplant in Seacroft, Leeds) in a 37°C incubator at 1% oxygen, 3% carbon dioxide, and 96% nitrogen gas mixture. Cultures were synchronized with 5% sorbitol (36). Synchronized cultures with a total volume of 18 mL at 1% parasitemia and 5% hematocrit were placed in nonvented 75 cm^2^ tissue culture flasks (Nunclon). Red blood cells at 5% hematocrit were used as control. One milliliter of culture was collected at time 0 and every 6 hours until 48 hours post synchronization. The collected sample was placed in a 1.5 mL microcentrifuge tube and centrifuged at 3,000 RPM for 2 minutes. The spent media were collected and stored at −80°C prior to analysis. Three biological replicates were analyzed.

A glucose assay on the spent media was performed using a glucose oxidase-peroxidase format assay kit (Megazyme). Amino acid concentrations in the spent media were determined using an Ultimate 3000 High-Performance Liquid Chromatography system (Dionex, UK). A ramp gradient reverse-phase chromatography was done with an Acclaim 120 C18 (Dionex, UK) 100 × 2.1 mm column (3 µm particle size, 120 Å pore size) stationary phase. Eluent A (10 mM Na_2_HPO_4_, 10 mM Na_2_HB_4_O_7_·10 H_2_O, 0.5 mM NaN_3_, pH 8.2) and eluent B (45% methanol [vol/vol] 45% acetonitrile [vol/vol] in water) constituted the mobile phase (Table S1).

Samples were derivatized manually within 10 minutes prior to chromatography. The derivatization procedure was done in a 1.5 mL microcentrifuge tube at room temperature by adding the following reagents in this particular order:

1. 300 µL of borate buffer (0.1 M Na_2_HB_4_O_7_·10H_2_O, pH 10.2)
2. 15 µL of o-phthaldialdehyde (OPA) (75 mM OPA, 225 mM 3-mercapto-propionic acid in 0.1 M borate buffer, pH 10.2)
3. 3 µL of sample (mixed five times using a Gilson pipette set at 300 µL)
4. 6 µL 9-fluorenylmethoxycarbonyl chloride (FMOC) solution (2.5 mg/mL FMOC in acetonitrile) (mixed five times using a Gilson pipette set at 300 µL)
5. 42 µL phosphoric acid solution (15 µL/mL 85% phosphoric acid in eluent A)

Detection of derivatized amino acids was through UV absorbance at 338 nm at 10.0 Hz data collection rate (from 0 to 55 minutes).

Spent media for both infected and uninfected (control) samples were assayed for glucose and amino acid concentrations as detailed above. The net metabolite flux from time 0 ($t_{0}$) to time *n* ($t_{n}$) was calculated using the formula:


$${flux}_{t_{0}-t_{n}}=\frac{v_{t_{0}}\times \left(∆C_{i}-∆C_{u}\right)}{{gDW}_{t_{0}}\times ∆t}$$

where

$v_{t_{0}}$= volume at $t_{0}$, in liters

$∆C_{i}$= change in metabolite concentration in infected culture from *t*_0_ to *t*_*n*_, in millimolars

$∆C_{u}$= change in metabolite concentration in uninfected culture from *t*_0_ to *t*_*n*_, in millimolars

${gDW}_{t_{0}}$= gram dry weight of parasite at $t_{0}$

∆t= change in time ($t_{n}-t_{0}$), in hours

The parasite mass ${gDW}_{t_{0}}$ was determined using the calculated number of parasites based on the parasitemia, hematocrit, and the volume at $t_{0}$:


$$numberofparasites=k\times p\times h\times v_{t_{0}}$$

where

k= hematocrit constant $\left(10^{13}\frac{RBCs}{litreofRBCs}\right)$

p= parasitemia at *t*_0_

h= hematocrit at *t*_0_

$v_{t_{0}}$= volume at *t*_0_, in liters

In this case, it was assumed for simplicity that each infected RBC contained only one parasite. The total parasite mass (gDW) was then calculated by multiplying the number of parasites by the mass per parasite experimentally measured previously $\left(10.5\times 10^{-12}\frac{gDW}{parasite}\right)$ (15). Flux calculations were done to represent the three main blood stage forms: the change in concentration from $t_{6}$ to $t_{18}$ was used to calculate the early to mid-ring stage flux, from $t_{30}$ to $t_{36}$ for the late trophozoite, and from $t_{42}$ to $t_{48}$ for the late schizont stage. Stage-specific average flux values ± standard deviations were used as upper and lower boundary flux constraints, respectively, for the corresponding boundary transport reaction. A positive flux represents the entry of metabolites into the model, while a negative flux represents the movement of metabolites into the external environment.

### Model simulations

To mimic *in vitro* consumption of nucleosides/bases from the media, the boundary flux of adenine, adenosine, guanine, and xanthine was set to zero, as these are not present in Albumax II supplemented with RPMI media. This allowed the model to consume hypoxanthine for purine metabolism from the external environment. Lower and upper bound flux values for the biomass reaction were set to 1 and 50 mmol/gDW/h, respectively. As for reversible metabolic reactions, the lower and upper bound flux values were set to −500 and 500 mmol/gDW/h, respectively. Nonreversible reactions were given either a lower bound or upper bound flux of zero depending on the direction of the reaction (Data S1).

Single-gene knockout simulation was done using COBRApy (37) with the “OBJECTIVE_COEFFICIENT” value = “1” assigned to the biomass reaction imposing the simulation to maximize the production of biomass (i.e., flux) with every knockout. A given gene knockout that produced less than 95% of the optimal biomass was identified as growth limiting, whereas a knockout that produced a zero biomass flux was considered lethal.

Model-predicted essential genes were compared against published data on 128 experimentally validated essential genes/reactions in *Plasmodium* (i.e., gold-standard list). Furthermore, gene essentiality data from the *Plasmodium* Genetic Modification (PlasmoGEM) database were also used to obtain essential genes that were added to the gold-standard list. PlasmoGEM, developed as part of the initiative of the Malaria Program at the Wellcome Trust Sanger Institute, is a database that holds prepublication phenotypic data on more than 2,000 *Plasmodium berghei* genes (37, 38). Updated *P. berghei* gene IDs along with phenotypic data were downloaded from the PlasmoGEM database, and orthologs of these genes in *P. falciparum* were identified through PlasmoDB (30). For *P. falciparum* genes that are orthologous to more than one *P. berghei* gene, only those with consistent gene essentiality information were noted (e.g., all orthologous *P. berghei* genes must be essential). A total of 1,169 orthologous genes were identified and used to validate the model-predicted essential genes (Table S2 to S4). The hypergeometric *P*-value was calculated to determine if the enrichment of experimentally validated essential genes/reactions in the list of model-predicted essential genes is statistically significant.

### Plasmid construction

The repair plasmid for disrupting the *UCK* gene was designed by inserting a *loxP* site upstream within intron 2 and another *loxP* site downstream at the end of the coding sequence. This strategy allows for excision of most of the active site upon rapamycin (RAP) induction, generating a conditional knockout for functional analysis of the gene. Two repair plasmids, namely pL6-UCK_loxP-sgRNA4-native E3-I3 and pL6-UCK_loxP-sgRNA4-native E3-ΔI3, were constructed in this study. Initially, the repair plasmid pL6-UCK_loxP-sgRNA4-native E3-I3 was created based on the plasmid pL6 eGFP (39), kindly provided by Prof. Jose-Juan Lopez-Rubio (Biology of Host-Parasite Interactions Unit, Institut Pasteur, Paris, France). This repair plasmid retains both exon 3 and intron 3 in their native sequence, while pL6-UCK_loxP-sgRNA4-native E3-ΔI3 was generated by removing the native intron 3 sequence from the former construct. Further details of the plasmid construction are provided in Data S2. For the sgRNA with NGG protospacer adjacent motif, it was designed using the sgRNA design tool (40).

### *P. falciparum* transfection and cloning

The DiCre-expressing parasite strain B11 (21) was cultured in supplemented RPMI1640 growth medium as above. The parasite cultures were incubated horizontally at 37°C with a low oxygen gas mixture as previously described (8). For synchronization, ring-stage parasites were generated by treating with 5% D-sorbitol for 10 minutes. To enrich the late stages (trophozoite and schizont) of *P. falciparum* parasites, a method based on the sedimentation behavior of late-stage parasites in gelatin solution, described by Ranford et al. (41) and Roncalés et al. (42) with minor modifications, was employed. Approximately 7%–8% parasitemia of asexual stages (mostly trophozoite-stage parasites) was pelleted using centrifugation at 250 × *g* for 10 minutes, and the supernatant was discarded. The pellet was then resuspended in prewarmed media at a 3:1 ratio (medium:pellet), followed by the addition of two volumes of gelatin solution (ZeptoGel; Gentaur). After gentle mixing, the solution was incubated at 37°C for 30 minutes. The supernatant containing the trophozoites and schizonts was transferred into a new culture flask, ready for transfection of DNA-loaded erythrocytes.

The transfection protocol followed the preloading of erythrocytes as described by Deitsch (43). Positive selection was carried out using 1 nM WR99210 (a gift from Jacobus Pharmaceuticals) to select for parasites carrying the *hDHFR* gene, the known target of WR99210 (44), and 250 nM DSM265 (synthesized by the Department of Chemistry, University of Leeds) to maintain Cas9 expression, as the episomal plasmid carries the yeast *dihydroorotate dehydrogenase* (*yDHODH*) gene, the target of DSM265 (45, 46). Drug selection began 48 hours after transfection and was maintained for 4 days. Upon detection of parasites via thin blood smearing, negative selection drug 40 µM 5-fluorocytosine was applied for 5 days to eliminate parasites carrying episomal plasmids. Subsequently, parasites were cloned using a limiting dilution method, aiming for a calculated dilution of 0.5 parasites per well with 3% hematocrit. Each well of 96‐well flat‐bottom plates contained 200 µL of the diluted parasite culture and was incubated in a humidified sealed chamber with low oxygen at 37°C. Parasite maintenance included changing the medium every other day, with daily flushing of cultures with low oxygen gas. Fresh RBCs were replenished every 5 days for parasite reinvasion. Samples were collected between days 10 and 14 for thin blood smear analysis. Subsequently, selected positive parasite clones were transferred to T25 flasks and cultured in complete RPMI 1640 media with 5% hematocrit.

### Conditional knockout induction

DiCre-driven *loxP* site recombination for gene function analysis was induced by treating tightly synchronized ring stage of transgenic parasite clones with 50 nM RAP (Stratech Scientific Ltd, Ely) or mock treatment using 0.1% (vol/vol) dimethyl sulfoxide (DMSO) for 24 hours. Transgenic parasite control F5, carrying a single *loxP* site, was used to control for unexcised parasites. At 42 hours post-RAP treatment, 200 µL of parasite cultures was collected for genomic DNA (gDNA) extraction by the microwave irradiation method (47). Next, the truncated *PfUCK* was assessed by PCR analysis of parasite gDNA using primers U6 (TCTACCTTTTAAAAAATAGCAAGCA) and P1 (CAAGTATATATTTTGTTTCTATAAATTGATATCTTA).

### Parasite growth assay

Parasite growth was determined in samples collected on days 0, 2, and 4 post-RAP treatment with parasitemia measured via fluorescence-activated cell sorting (FACS) as previously described (16). Parasite samples were stained with SYBR Green I nucleic acid gel stain (Thermo Fisher Scientific, UK) by the following procedure: initially, 100 µL of parasite cultures was centrifuged at 800 × *g* for 1 minute. Subsequently, the cell pellets were washed with 500 µL of phosphate-buffered saline (PBS) and centrifuged again. The parasite samples were then fixed with 100 µL of 4% paraformaldehyde (Thermo Fisher Scientific, UK) and 0.1% glutaraldehyde (Sigma-Aldrich, USA) in PBS for 1 hour at 4°C, followed by another PBS wash. Next, the cells were stained with 1:5,000 SYBR Green I in PBS for 1 hour at 37°C, washed with PBS, and resuspended in 100 µL of PBS. Finally, 10 µL of stained cells was diluted into 1 mL of PBS in an Eppendorf tube and analyzed using a CytoFLEX S Flow Cytometer with a 525/40 BP filter to determine the proportion of SYBR Green-stained cells per 100,000 cells. Statistical analysis of the growth assay was conducted using GraphPad Prism 9 with a two-way ANOVA analysis (Bonferroni’s multiple comparison test), with results indicating statistical significance for *P <* 0.05.

### Gene expression quantitation

Total RNA was isolated from parasite pellets utilizing the Direct-zol RNA MiniPrep Kit (Zymo Research, USA) in accordance with the manufacturer’s instructions. Subsequently, genomic DNA was eliminated from RNA samples using the Turbo DNA-Free Kit (Invitrogen, catalog number AM1907). Then, 120 ng of DNA-free RNA samples served as the template for cDNA synthesis employing the Maxima H Minus First Strand cDNA Synthesis Kit (Thermo Fisher Scientific, catalog number K1651), with oligo (dT)18 acting as the reverse transcription primer. For RT-qPCR reactions, the Brilliant III Ultra-Fast QPCR Master Mix (Agilent Technologies, USA, catalog number 600880) was utilized. Amplification of the *PfUCK* gene was carried out using the C3F2 forward primer (5′-TGAGGATTGTATAAACAACGGTAAAATCGTGCCGG-3′) and C3F2 reverse primer (5′-TTTTTTTTCAACACCAGATTCACCCTGGTCGTT-3′), while the small subunit rRNA (SSU rRNA) reference gene was amplified using the ssu_q_forward (5′-CGAACGAGATCTTAACCTGC-3′) and ssu_q_reverse (5′-CACACTGTTCCTCTAAGAAGC-3′) primers. RT-qPCR was initiated with an initial denaturation step at 95°C for 2 minutes, followed by 40 cycles of denaturation at 95°C for 30 seconds, annealing at 65°C for 30 seconds, and extension at 68°C for 30 seconds, carried out in a C100 Thermo Cycler (Bio-Rad, USA) with default settings. Thermal melt assay revealed single amplicons of the anticipated size. Relative changes in gene expression were determined using the 2^−ΔΔCT^ method (48). Statistical analysis using two-tailed Student’s *t*-tests was performed to assess the significance of changes in mRNA levels as unpaired samples.

### UCK protein expression and purification

The native PfUCK protein has a hydrophobic region spanning amino acid residues 8–23 as predicted by the Malaria Secretory Signal Predictions (MalSig) program (49). The hydrophobic core at the protein’s N-terminus may cause poor expression and low solubility in *E. coli*. Therefore, a truncated gene was created where the ORF began at amino acid residues 24–371, encoding a 348 amino acid protein. Subsequently, the optimized codons for recombinant expression of the truncated PfUCK were generated using the program OPTIMIZER (50), then were commercially synthesized and cloned into the plasmid pUC57 (GenScript). Using a pair of primers (5′-TGCCGCGCGGCAGCCATATGGAAAACTTCTACCTGCTG-3′ and 5′-TCGAGTGCGGCCGCAAGCTTTTACATGTTGGAAAACG-3′), the truncated gene was amplified from the plasmid pUC57 and subsequently subcloned into the pET28a expression vector via *Nde*I and *Hind*III cloning sites. The pET28a plasmid containing *PfUCK* was transformed into *E. coli* BL21 (DE3) Rosetta cells, which were then selected and cultured for subsequent protein purification. Briefly, protein expression was induced with isopropyl-D-thiogalactoside, incubated at 16°C with shaking at 200 RPM for 20 hours, and the cell pellet resuspended in binding buffer (50 mM Tris [pH 7.6], 300 mM NaCl, 20 mM imidazole, 5% glycerol, and 0.075% β-mercaptoethanol), with cell disruption using an Avestin C3 Cell Disrupter. Cell debris was removed, and the UCK was purified by Ni-NTA affinity purification. The protein was eluted from the column using a linear gradient of imidazole (20–500 nM) in elution buffer (50 mM Tris [pH 7.6], 300 mM NaCl, 5% glycerol, and 0.075% β-mercaptoethanol). PfUCK-containing fractions were combined and concentrated before assessing purity via 12% SDS-PAGE. The concentrated protein was then stored at −70°C in a storage buffer (25 mM Tris-HCl, 50 mM KCl, and 20% glycerol; pH 7.0).

Recombinant human UMP-CMP kinase (hUCK) was constructed by amplifying its ORF from an available commercial plasmid (CMPK1 [NM_016308] Human Tagged ORF Clone–RC204856; OriGene) using primers hUCK forward (5′-TGCCGCGCGGCAGCCATATGATGCTGAGCCGC-3′) and reverse (5′-TCGAGTGCGGCCGCAAGCTTTTAGCCTTCCTTGTCAAAAAT-3′). The gene was cloned into the pET28a expression plasmid, and hUCK expressed in induced BL21 (DE3) cells that contain extra copies of the *argU*, *ileY,* and *leuW* tRNA genes similar to PfUCK. The protein was purified with a HisPur Ni-NTA Spin column, and elution was performed with 250 mM imidazole in elution buffer (50 mM Tris-HCl, 750 mM NaCl, and 10% glycerol; pH 7.5). The protein was further purified using a His GraviTrap TALON column (Cytiva, catalog number 29-0005-94). The buffers employed were binding buffer (50 mM potassium phosphate [KP], 100 mM NaCl, and 10% glycerol; pH 8.0), washing buffer (50 mM KP, 500 mM NaCl, 10 mM imidazole, and 10% glycerol; pH 8.0), and elution buffer (50 mM Tris-HCl, 750 mM NaCl, 250 mM imidazole, and 10% glycerol; pH 7.5). The purified protein was desalted using a PD-10 column (Sephadex G25 M; Cytiva, catalog number 17085101) stored in a storage buffer (25 mM Tris-HCl, 50 mM KCl, and 20% glycerol; pH 7.0). Purity of the recombinant protein was verified via SDS-PAGE (Mini-Protein TGX Precast Gels; Bio-Rad), and its concentration was determined using a Bradford protein assay (Bio-Rad, catalog number 5000006).

cells contain extra

copies of the *argU*, *ileY,* and *leuW* tRNA gene

similar to PfUCK. The protein was purified with a HisPur Ni-NTA Spin column, and elution was performed with 250 mM imidazole in elution buffer (50 mM Tris-HCl, 750 mM NaCl, and 10% glycerol; pH 7.5). The protein was further purified using a His GraviTrap TALON column (Cytiva, catalog number 29-0005-94). The buffers employed were binding buffer (50 mM potassium phosphate [KP], 100 mM NaCl, and 10% glycerol; pH 8.0), washing buffer (50 mM KP, 500 mM NaCl, 10 mM imidazole, and 10% glycerol; pH 8.0), and elution buffer (50 mM Tris-HCl, 750 mM NaCl, 250 mM imidazole, and 10% glycerol; pH 7.5). The purified protein was desalted using a PD-10 column (Sephadex G25 M; Cytiva, catalog number 17085101) stored in a storage buffer (25 mM Tris-HCl, 50 mM KCl, and 20% glycerol; pH 7.0). Purity of the recombinant protein was verified via SDS-PAGE (Mini-Protein TGX Precast Gels; Bio-Rad), and its concentration was determined using a Bradford protein assay (Bio-Rad, catalog number 5000006).

### UCK characterization

Enzyme activity was assessed using an indirect spectrophotometric assay that measured NADH absorbance at 340 nm coupled to pyruvate kinase/lactate dehydrogenase. The reaction mixture contained 50 mM Tris-HCl (pH 7.6), 50 mM KCl, 10 mM MgCl_2_, 5 mM ATP, 0.2 mM NADH, 1 mM phosphoenolpyruvate, 10 mM dithiothreitol, pyruvate kinase (10 U/mL), lactate dehydrogenase (15 U/mL), and purified UCK (OD340/min of 0.04–0.06). The reaction was initiated by adding 1 mM of the substrate (CMP, UMP, or dCMP), and the assay was performed at 25°C for 10 minutes in the absence or the presence of UCK. The decrease in absorbance was monitored using a UV/visible spectrophotometer (Ultrospec 2100 Pro).

UCK’s kinetic properties were determined by varying nucleoside monophosphate (CMP, UMP, and dCMP) concentrations. Then, a reaction rate was calculated from the decrease in absorbance at 340 nm (ε_NADH_ = 6.22 × 10^−3^ M^−1^ cm^−1^) compared to the control reaction in which UCK was absent. The maximum reaction rate (*V*_max_) and Michaelis constant (*K*_*m*_) values were calculated using the Michaelis-Menten equation (*v* = *V*_max_[*S*] /*K*_*m*_+ [*S*]), where *v* is the reaction rate and [*S*] is the substrate concentration.

### Inhibition constant (*K*_*i*_) determination

The 1 mL reaction assay consisted of 50 mM Tris-HCl (pH 7.6), 50 mM KCl, 10 mM MgCl_2_, 5 mM ATP, 0.2 mM NADH, 1 mM phosphoenolpyruvate, 10 mM DTT, pyruvate kinase (10 U/mL), lactate dehydrogenase (15 U/mL), and appropriate amounts of purified UCK (yielding an OD340/min of 0.04–0.06) along with varying concentrations of the inhibitor. The final concentration of dimethyl sulfoxide (Merck, Catalog number 472301) in the assay reaction was maintained at 1%. Compounds were initially screened for inhibition of recombinant PfUCK enzyme activity using a 10-fold serial dilution in DMSO. Active compounds were then further tested in a twofold serial dilution to determine their *K*_*i*_ values. The enzyme assay was as described above, with initiation by adding 1 mM CMP. As inhibitors were obtained from a virtual screening using the model structure of PfUCK as the template, *K*_*i*_ values for each inhibitor were determined using nonlinear regression curve fitting in Prism software based on a competitive equation utilizing CMP’s *K*_*m*_ value for calculation. Using the competitive equation here is based on an assumption that those inhibitors bind the active site of a protein. Compounds active against PfUCK were also tested for their *K*_*i*_ values against hUCK using the same protocol as for PfUCK.

### Antiplasmodial activity (IC_50_) determination

*P. falciparum* 3D7 parasites underwent two synchronization cycles with D-sorbitol initiating the inhibitor testing. Parasitemia and hematocrit were adjusted to 0.5% and 3%, respectively. Synchronized ring parasites were cultured in 96-well plates in complete RPMI medium with varying inhibitor concentrations. Inhibitor stock solutions, mostly at 100 mM, were prepared in DMSO. However, some compounds had 50 mM stock solutions due to solubility limitations. Triplicate wells were prepared for each compound. Negative controls included wells with uninfected red cells (3% hematocrit) in complete RPMI medium.

Culture plates were incubated in a chamber with controlled gas conditions at 37°C. After 48 hours, parasites were quantified using a fluorescence-based assay with SYBR Green dye as previously described (51). Lysis buffer containing SYBR Green was added to each well, and plates were incubated at room temperature for 45 minutes and protected from the light. Fluorescence was measured using a microplate reader (POLARstar OPTIMA, BMG Labtech) with excitation at 485 nm and detection at 520 nm. Background fluorescence of uninfected red cells was subtracted, and dose-response curves were plotted against inhibitor concentration. IC_50_ values were determined using nonlinear regression curve fitting in GraphPad Prism software.

## RESULTS

### Development of a combined genome-scale metabolic model

Three malaria metabolic models developed by Forth (15), Plata et al. (27), and Huthmacher et al. (14) were combined to develop a highly curated genome-scale metabolic model supported by experimentally derived metabolite data. Having the fewest dead-end metabolites and reactions, the Forth model was used as the base model, and reactions from the two other models were added in an iterative manner (i.e., reassessing after every addition of reaction). This method ensured no additional dead ends were incorporated into the final model while maximizing the total number of reactions contributed by the Plata et al. (27) and Huthmacher et al. (14) models. The final model, iFT342, has a total of 342 genes, 551 reactions, and 560 metabolites. The model includes five compartments: the apicoplast, cytosol, mitochondria, vacuole, and the external compartment. Out of the 551 reactions in the model, 371 (67.3%) are known to be facilitated by an enzyme/protein associated to a specific *P. falciparum* gene (i.e., gene associated). Most of these gene-associated reactions are intracellular reactions. The rest of the reactions that do not have associated genes were transport reactions (91, 16.5%) to facilitate movement of metabolites from one compartment to another, and boundary reactions (74, 13.4%) allow metabolites to enter the parasite from the external compartment, while only 15 (2.7%) are intracellular reactions. The model has a biomass reaction that represents the utilization of different components necessary for parasite growth.

**Fig 1:**
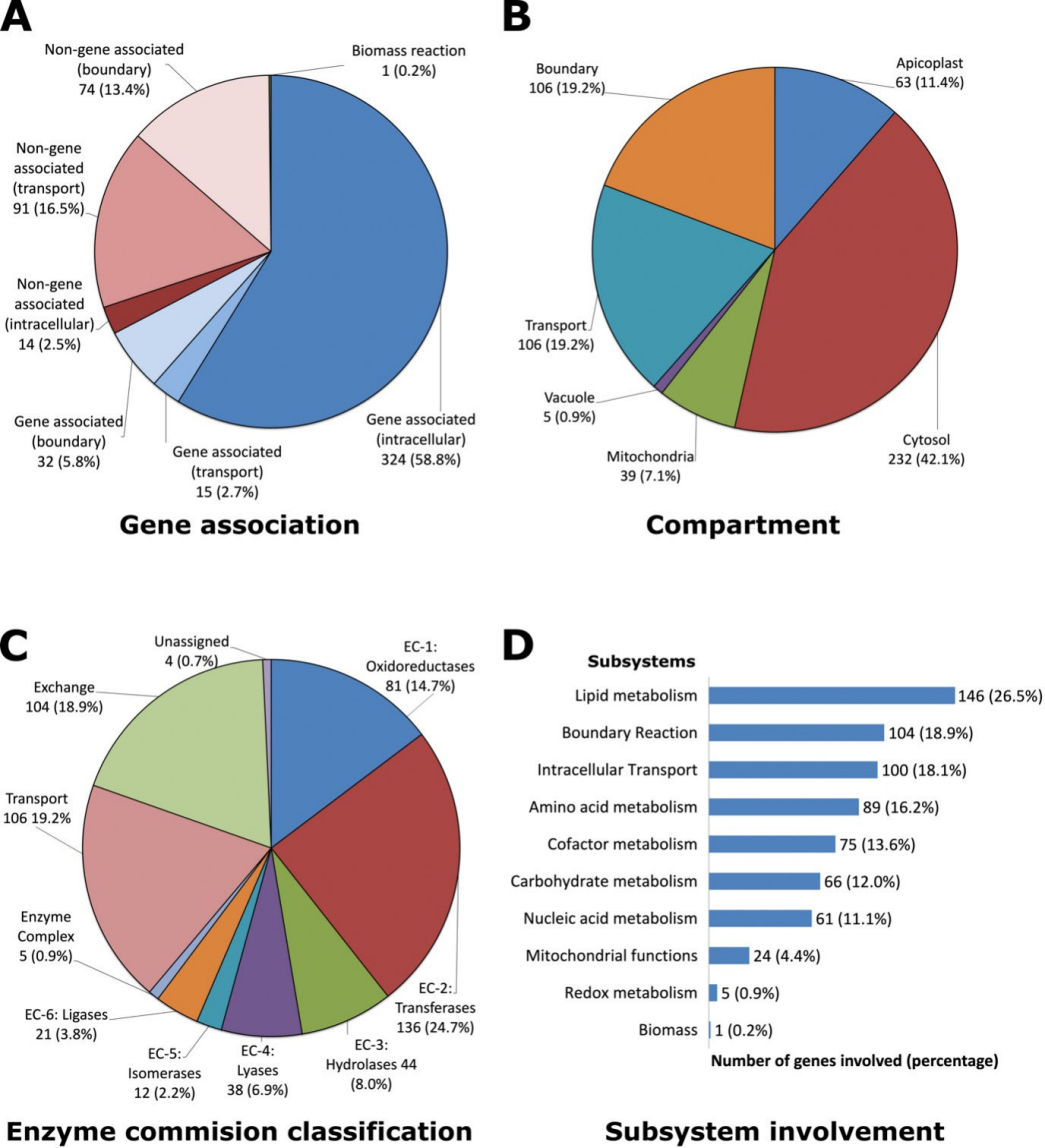
Reactions in the iFT342 model. Reactions that comprise the iFT342 *P. falciparum* genome-scale metabolic model have been grouped according to (**A**) gene association, (**B**) compartment localization, (**C**) enzyme commission classification, and (**D**) subsystem involvement. *In vitro* flux of glucose and 18 amino acids was measured in highly synchronized *in vitro* cultured *P. falciparum* 3D7 at three different time points to represent the early to mid-ring trophozoite stages, late trophozoite stages, and late schizont stages (Table S5). In addition, measurements of lactate in spent media from previous experimental data conducted in our facility were incorporated into the model (15). These flux values were incorporated into the model to generate stage-specific models. The standard deviation above and below the calculated mean flux was assigned as the upper and lower bound constraints of the corresponding boundary reaction in the model, respectively.

Out of a total of 560 metabolites in the model, 106 are boundary metabolites, while 454 are intracellular metabolites. All metabolites in the model have chemical formulas, and 530 have additional metabolite attributes, i.e., PubChem ID (52), IUPAC International Chemical Identifier (InChI) keys (53), and canonical Simplified Molecular-Input Line-Entry System (54). Metabolite information, reaction stoichiometries with their corresponding lower and upper flux bounds, and the final biomass composition are all encoded into the final model (Data S1). Figure 1 shows the distribution of reactions based on the gene association, compartment, EC classification, and subsystem involvement. The four reactions that fall under the unassigned category (Fig. 1C) include (a) hemoglobin oxygenation, (b) combined pyridoxine biosynthesis protein (PDX1 and PDX2) reaction, (c) spontaneous L-glutamate 5-semialdehyde to (S)-1-pyrroline-5-carboxylate reaction, and (d) the biomass reaction.

### Genome-scale modeling asserts a possible antimalarial target

COBRApy (55) was used to perform single-gene knockout simulations on 342 genes in the model (Fig. S1; Table S6). This was done to identify essential genes in the schizont stage of the model. Using this approach, 87 genes were predicted to be essential, of which 47 gene knockouts were predicted to be lethal (i.e., biomass solution = 0) and 40 gene knockouts were growth-limiting (i.e., biomass solution <95% optimal biomass solution). The list of 87 genes was compared to a list of experimentally validated essential genes as described in the Materials and Methods section. We have observed a statistically significant increase (enrichment of 1.51) in the percentage of experimentally validated essential genes within the pool of model-predicted essential genes (58 out of 87, 66.7%) in comparison with the percentage of experimentally validated essential genes in the whole model (151 out of 342, 44.2%; hypergeometric *P*-value = 6.41 × 10^−7^). Interestingly, the 40 predicted growth-limiting essential genes are all found in the experimentally validated essential gene list (enrichment of 2.26; hypergeometric *P*-value = 2.29 × 10^−16^).

The model also predicted 18 novel *P. falciparum* essential genes that are not in the gold-standard list (Table 1). These assert genes whose products are potential antimalarial drug targets. As the majority of antimalarial drugs inhibit enzymes conserved in humans but targeting nonconserved residues, these have potential as drug targets. Modeling predicts that UCK plays a crucial role in *P. falciparum* pyrimidine metabolism, converting UMP, CMP, and dCMP into their respective diphosphate forms (UDP, CDP, and dCDP). These diphosphate forms are further metabolized into triphosphates (UTP, CTP, and dCTP), essential for DNA and RNA synthesis (56). Notably, *P. falciparum* relies solely on the *de novo* pathway for pyrimidine nucleotide biosynthesis, unlike human cells that utilize both salvage and *de novo* pathways (57). Additionally, UCK’s medicinal importance lies in its involvement in phosphorylating drug precursors like gemcitabine, troxacitabine, and lamivudine to their active forms, used for treating cancer and viral infections (58–64).

**TABLE 1:** List of novel gene targets and their associated protein/enzyme reactions predicted as lethal or growth limiting by the schizont-stage model of the iFT342 genome-scale metabolic model^*a*^

Gene ID	Associated reaction/s	Pathway/s	EC number	Predicted effect of gene knockout
PF3D7_0111500	UMP-CMP kinase	Pyrimidine Metabolism	2.7.4.14	Lethal
PF3D7_0204500	Aspartate aminotransferase	Pyruvate metabolism, glutamate metabolism, asparagine and aspartate metabolism	2.6.1.1	Growth limiting
PF3D7_0507200	Oxyhemoglobin digestion: vacuole	Hemoglobin digestion	3.4.11.- and 3.4.11.1 and 3.4.11.2 and 3.4.11.9 and 3.4.11.18 and 3.4.11.21 and 3.4.14.1 and 3.4.21.62 and 3.4.22.- and 3.4.23.- and 3.4.23.38 and 3.4.23.39 and 3.4.24.-	Growth limiting
PF3D7_0616000	Pyridoxal kinase and pyridoxal phosphatase	Vitamin B6 metabolism	2.7.1.35	Growth limiting
PF3D7_0801800	Mannose-6-phosphate isomerase	Mannose and fructose metabolism	5.3.1.8	Lethal
PF3D7_0813800	GDP-mannose 4,6-dehydratase	Mannose and fructose metabolism	4.2.1.47	Lethal
PF3D7_0815900	2-Oxoglutarate dehydrogenase complex	Mitochondrial TCA cycle	1.2.4.2 and 1.8.1.4 and 2.3.1.61	Lethal
PF3D7_0820700	2-Oxoglutarate dehydrogenase complex	Mitochondrial TCA cycle	1.2.4.2 and 1.8.1.4 and 2.3.1.61	Growth limiting
PF3D7_0823900	Citrate transfer: mitochondria to cytosol; dicarboxylate/tricarboxylate carrier	Mitochondrial TCA cycle, pyruvate metabolism, intracellular transport	Unknown	Growth limiting
PF3D7_0922600	L-glutamate ammonia ligase	Glutamate metabolism, nitrogen metabolism	6.3.1.2	Growth limiting
PF3D7_0927300	Fumarate hydratase	Mitochondrial TCA cycle	4.2.1.2	Growth limiting
PF3D7_0928900	Guanylate kinase	Purine metabolism	2.7.4.8	Lethal
PF3D7_0932300	Oxyhemoglobin digestion: vacuole	Hemoglobin digestion	3.4.11.- and 3.4.11.1 and 3.4.11.2 and 3.4.11.9 and 3.4.11.18 and 3.4.11.21 and 3.4.14.1 and 3.4.21.62 and 3.4.22.- and 3.4.23.- and 3.4.23.38 and 3.4.23.39 and 3.4.24.-	Growth limiting
PF3D7_1014000	GDP-L-fucose synthase	Mannose and fructose metabolism	1.1.1.271	Lethal
PF3D7_1017400	D-Mannose 6-phosphate 1,6-phosphomutase	Mannose and fructose metabolism	5.4.2.8	Lethal
PF3D7_1020800	Dihydrolipoamide acyltransferase component E2	Pyruvate metabolism	2.3.1.12	Lethal
PF3D7_1108500	Succinate-CoA ligase (ADP forming)	Mitochondrial TCA cycle	6.2.1.5	Growth limiting
PF3D7_1115300	Oxyhemoglobin digestion: vacuole	Hemoglobin digestion	3.4.11.- and 3.4.11.1 and 3.4.11.2 and 3.4.11.9 and 3.4.11.18 and 3.4.11.21 and 3.4.14.1 and 3.4.21.62 and 3.4.22.- and 3.4.23.- and 3.4.23.38 and 3.4.23.39 and 3.4.24.-	Growth limiting
PF3D7_1115400	Oxyhemoglobin digestion: vacuole	Hemoglobin digestion	3.4.11.- and 3.4.11.1 and 3.4.11.2 and 3.4.11.9 and 3.4.11.18 and 3.4.11.21 and 3.4.14.1 and 3.4.21.62 and 3.4.22.- and 3.4.23.- and 3.4.23.38 and 3.4.23.39 and 3.4.24.-	Growth limiting
PF3D7_1212000	Glutathione:hydrogen-peroxide oxidoreductase	Glutathione metabolism	1.11.1.9	Growth limiting
PF3D7_1251300	ATP:dUMP phosphotransferase	Pyrimidine metabolism	2.7.4.9	Lethal
PF3D7_1320800	2-Oxoglutarate dehydrogenase complex	Mitochondrial TCA cycle	1.2.4.2 and 1.8.1.4 and 2.3.1.61	Growth limiting
PF3D7_1368700	Mitochondrial carrier protein	Mitochondrial TCA cycle, intracellular transport	Unknown	Growth limiting
PF3D7_1401300	Oxyhemoglobin digestion: vacuole	Hemoglobin digestion	3.4.11.- and 3.4.11.1 and 3.4.11.2 and 3.4.11.9 and 3.4.11.18 and 3.4.11.21 and 3.4.14.1 and 3.4.21.62 and 3.4.22.- and 3.4.23.- and 3.4.23.38 and 3.4.23.39 and 3.4.24.-	Growth limiting
PF3D7_1431600	Succinate-CoA ligase (ADP forming)	Mitochondrial TCA cycle	6.2.1.5	Growth limiting
PF3D7_1437700	Succinate-CoA ligase (ADP forming)	Mitochondrial TCA cycle	6.2.1.5	Growth limiting
PF3D7_1446400	Dihydrolipoamide acyltransferase component E2	Pyruvate metabolism	2.3.1.12	Lethal
PF3D7_1453500	NADPH:NAD + oxidoreductase	Nicotinate and nicotinamide metabolism	1.6.1.1	Growth limiting
PF3D7_1459700	Pyridoxal 5-phosphate synthase	Vitamin B6 metabolism	1.4.3.5	Growth limiting

^*a*^ Single-gene knockout simulations using COBRApy were done while setting the model objective to maximize the biomass output. Biomass output of zero for a given gene knockout is considered lethal, while output of <95% of the optimal biomass solution is considered growth limiting. The table also includes metabolic pathways and enzyme commission number/s of the associated reaction/s.

### Strategy for generation of inducible UCK knockout mutants

**Fig 2:**
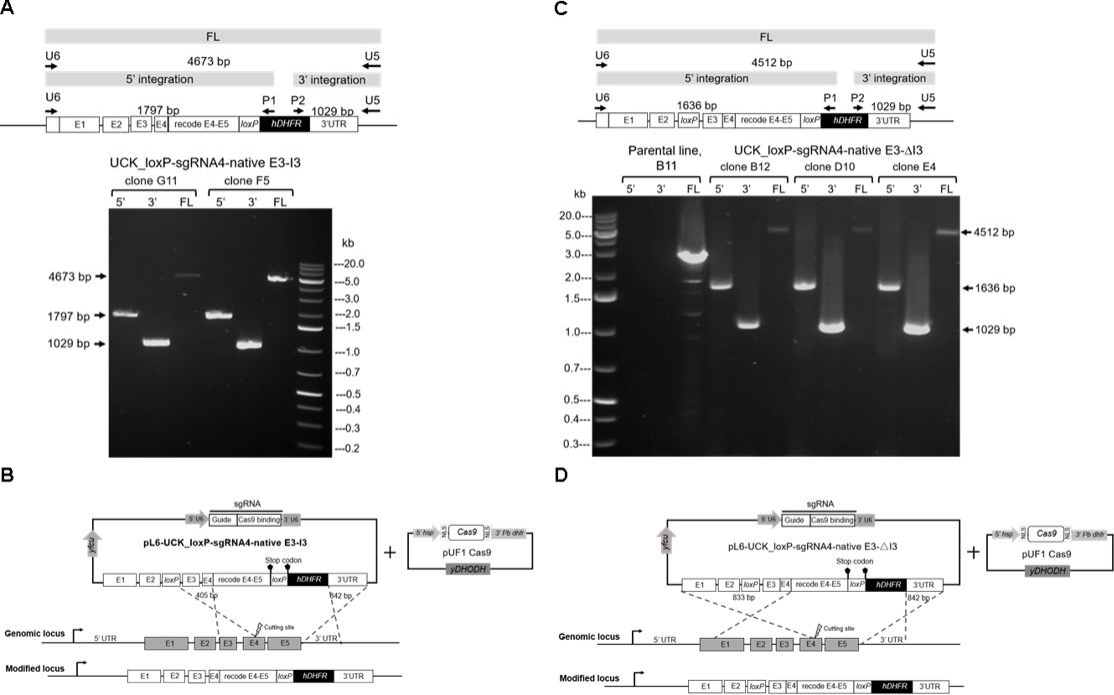
Diagnostic PCR analysis and homologous recombination scheme. (**A**) Diagnostic PCR analysis of genomic DNA from integrant *P*. *falciparum* clones (G11 and F5) obtained from transfections using the repair plasmid pL6-UCK_loxP-sgRNA4-native E3-I3. Both clones exhibited positive genotyping for 5′ and 3′ integration as well as the full length (FL) of the modified *PfUCK* locus, whereas DNA sequencing analysis of transfectant clone F5 shows the absence of the upstream *loxP* sequence. (**B**) Diagram illustrating a possible homologous recombination event in the transfectant clone F5, resulting in the integration of only the downstream *loxP* site into the target locus. (**C**) Diagnostic PCR analysis of genomic DNA from integrant *P*. *falciparum* clones (B12, D10, and E4) derived from transfections using the repair plasmid pL6-UCK_loxP-sgRNA4-native E3-ΔI3. These clones exhibited positive genotyping for 5′ and 3′ integration as well as the complete modification of the *PfUCK* locus. (**D**) Diagram illustrating the predicted homologous recombination event in transfectants of pL6-UCK_loxP-sgRNA4-native E3-ΔI3.

As attempts to recover viable parasites with disruptions in the *PfUCK* coding region in *P. falciparum* were unsuccessful (unpublished observation), we developed a strategy for generation of inducible *UCK* knockout mutants. Two repair plasmids with *loxP* sites inserted in introns, namely pL6-UCK_loxP-sgRNA4-native E3-I3 and pL6-UCK_loxP-sgRNA4-native E3-ΔI3, were designed that permit inducible deletion of the UCK enzyme’s active site upon induction (Fig. 2). Both plasmids were designed to induce a modified parasite, with an upstream *loxP* site strategically positioned in intron 2 and a downstream *loxP* site located at the end of the *UCK* gene. The plasmid pL6-UCK_loxP-sgRNA4-native E3-I3 retained exon 3 and intron 3 in their native sequence, while pL6-UCK_loxP-sgRNA4-native E3-ΔI3 was derived by eliminating the native intron 3 sequence from pL6-UCK-sgRNA4-native E3-I3.

Mutant parasites were generated by transfecting the repair plasmids into the DiCre-expressing *P*. *falciparum* B11 line (21). Successful modification of the *PfUCK* gene in the transfected parasite population was confirmed through diagnostic PCR. Subsequent limiting dilution cloning resulted in the generation of parasite clones G11 and F5 using the repair plasmid pL6-UCK_loxP-sgRNA4-native E3-I3 (Fig. 2A). Sequencing analysis of pL6-UCK_loxP-sgRNA4-native E3-I3 transfectant clone F5 revealed integration of only the downstream *loxP* site into the target locus, while the upstream *loxP* site was absent, potentially indicating alternative recombination during the repair process. Since this repair plasmid was designed to retain the native exon 3 and intron 3, a sequence of 405 bp between native exons 3 and 4 was preserved, which is sufficient for homologous recombination. Previous studies have shown that homologous regions of >250–1,000 bp are adequate for homology-directed repair in *P. falciparum* (65). Therefore, the 405 bp segment, situated close to the Cas nuclease cleavage site, exhibits potential for homologous recombination, as illustrated in Fig. 2B.

The transfectant parasites of pL6-UCK_loxP-sgRNA4-native E3-ΔI3, isolated as clones B12, D10, and E4, exhibited positive genotyping for 5′ and 3′ integration and complete modification of the *PfUCK* locus (Fig. 2C). DNA sequencing of transfectant clone E4 confirmed the successful integration of both upstream and downstream *loxP* sequences at the target *PfUCK* locus. This indicates the generation of transgenic parasites through the expected homologous recombination, as depicted in Fig. 2D.

### Generation of UCK knockout mutants

The insertion of the two *loxP* sites allows truncation of *UCK* by inducing DiCre expression. Hence, the clones with complete integration (referred to as UCK-clone B12, D10, and E4) were induced to delete the majority of the *UCK* gene (Fig. 2C and D). As a control, the clone F5 that harbors a single *loxP* site positioned at the end of the *UCK* gene was used. This parasite clone was designated as UCK-control F5 and served as a control for unexcised parasites following rapamycin treatment.

**Fig 3:**
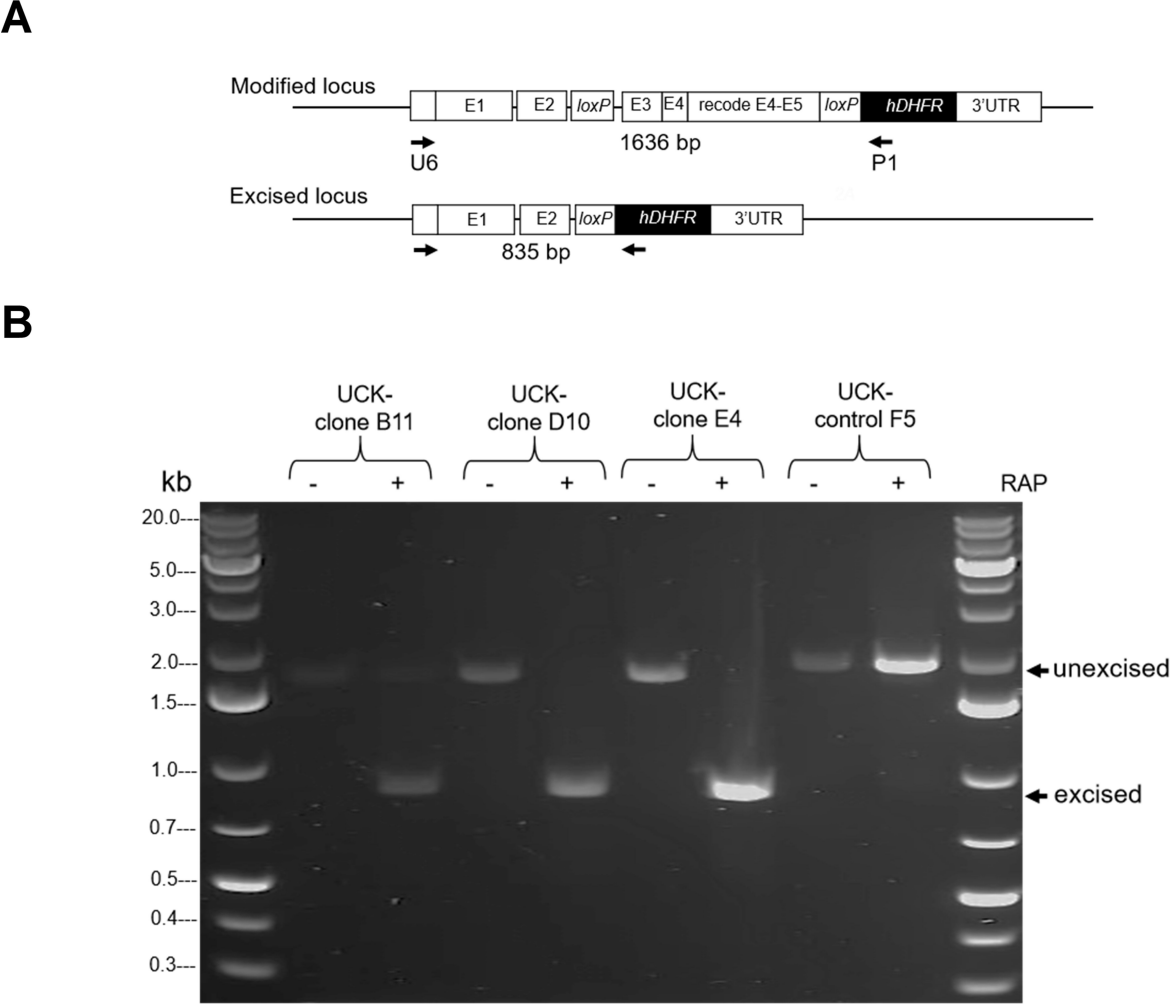
DiCre-mediated conditional disruption of *PfUCK.* (**A**) An illustration of the primers specifically designed for the detection of *PfUCK-*truncated parasites. (**B**) PCR products of mutants following RAP induction of cre/lox deletion. At 42 hours post-RAP treatment, 200 µL of parasite cultures was harvested for gDNA extraction and PCR analysis of DiCre-mediated excision of the *loxP*-floxed DNA. The expected sizes of the PCR amplicons, including unexcised and excised bands, are indicated by arrows.

To induce *UCK* gene truncation, tightly synchronized ring-stage transgenic parasite clones were subjected to treatment with either 50 nM RAP or a mock treatment using 0.1% DMSO for 24 hours. Expected site-specific recombination between the introduced *loxP* sites in the modified *PfUCK* locus of clones B12, D10, and E4 occurred upon RAP treatment, resulting in the reconstitution of a single *loxP*-containing parasite (excised locus; Fig. 3A). Diagnostic PCR results revealed a 1,636 bp band in DMSO-treated samples and an 835 bp band in RAP-treated samples, confirming the anticipated excision event. Conversely, only unexcised bands were amplified as expected in parasite UCK-control F5. Notably, unexcised DNA was detected in UCK-clone B12 (Fig. 3B).

### Phenotype of parasite UCK-induced knockouts

**Fig 4:**
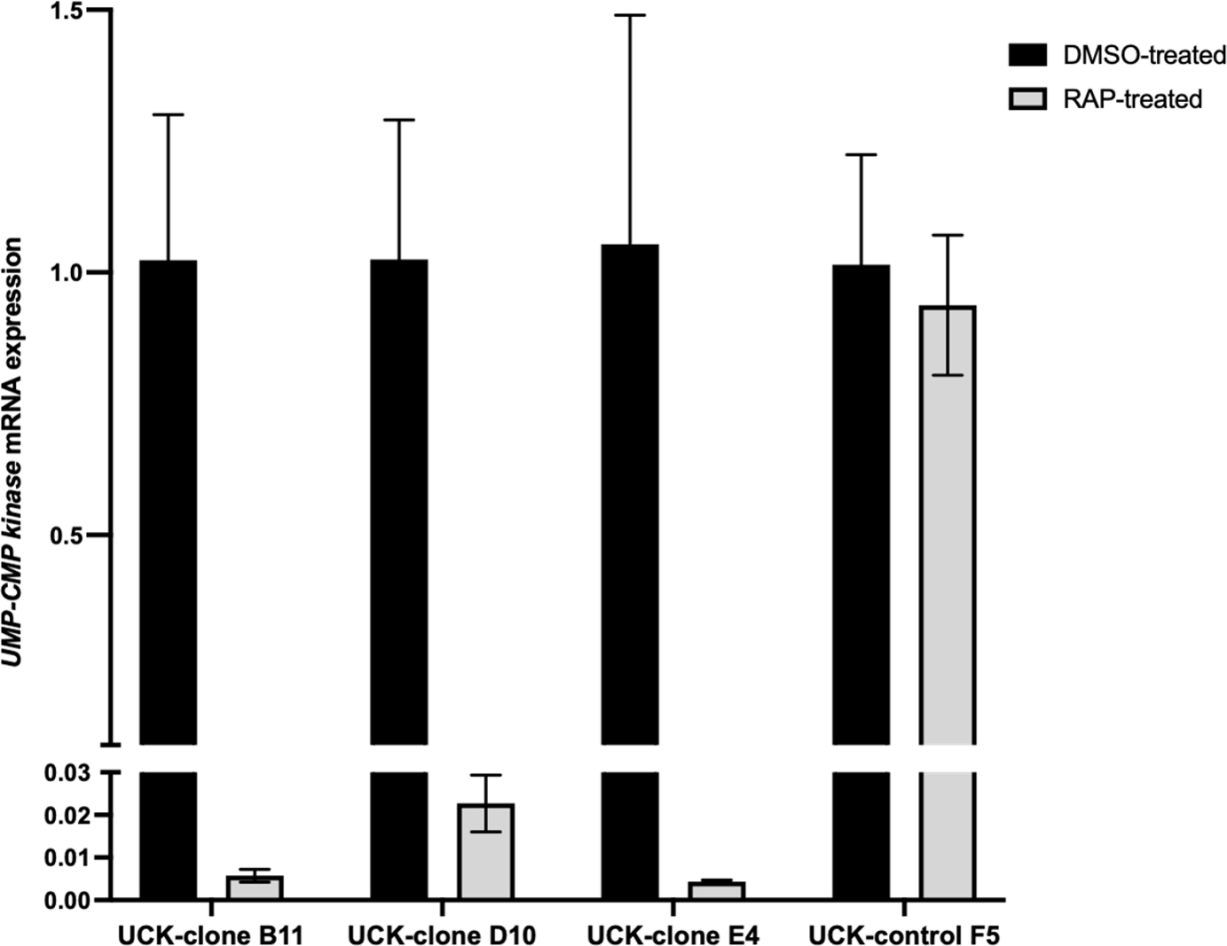
Analysis of *PfUCK* mRNA expression in response to RAP. RNA from rapamycin-treated parasites or placebo control was isolated from the transgenic clones and subjected to RT-qPCR for *PfUCK*. Transgenic parasite F5 (bearing a single *loxP* site) was used as the control for *PfUCK* gene expression. The levels of *PfUCK* mRNA in RAP-treated cultures relative to DMSO-treated cultures were determined by RT-qPCR using the 2^−ΔΔCT^ method by normalizing to *SSU* rRNA. Error bars represent the standard error of the mean across three replicates. Statistical analysis using two-tailed Student’s *t*-tests was performed to assess the significance of changes in mRNA levels as paired samples. NS denotes not significant, and a *P* < 0.05 was considered statistically significant with ***, *P* < 0.0001; **, *P*<0.001.

Since maximal expression of *UCK* is observed in trophozoites and persists into the schizont stage (30), trophozoite-stage parasites were selected as the source for studying gene expression levels, comparing untreated and RAP-treated parasites. Levels of *PfUCK* mRNA in UCK-clones B12, D10, and E4 were significantly reduced (approximately 45–230-fold) following RAP treatment (incubation with 50 nM RAP for 24 hours), based on qRT-PCR (Fig. 4). However, *PfUCK* mRNA levels in the control F5 were unaffected by RAP treatment.

### Dependence of parasite growth on UCK

**Fig 5:**
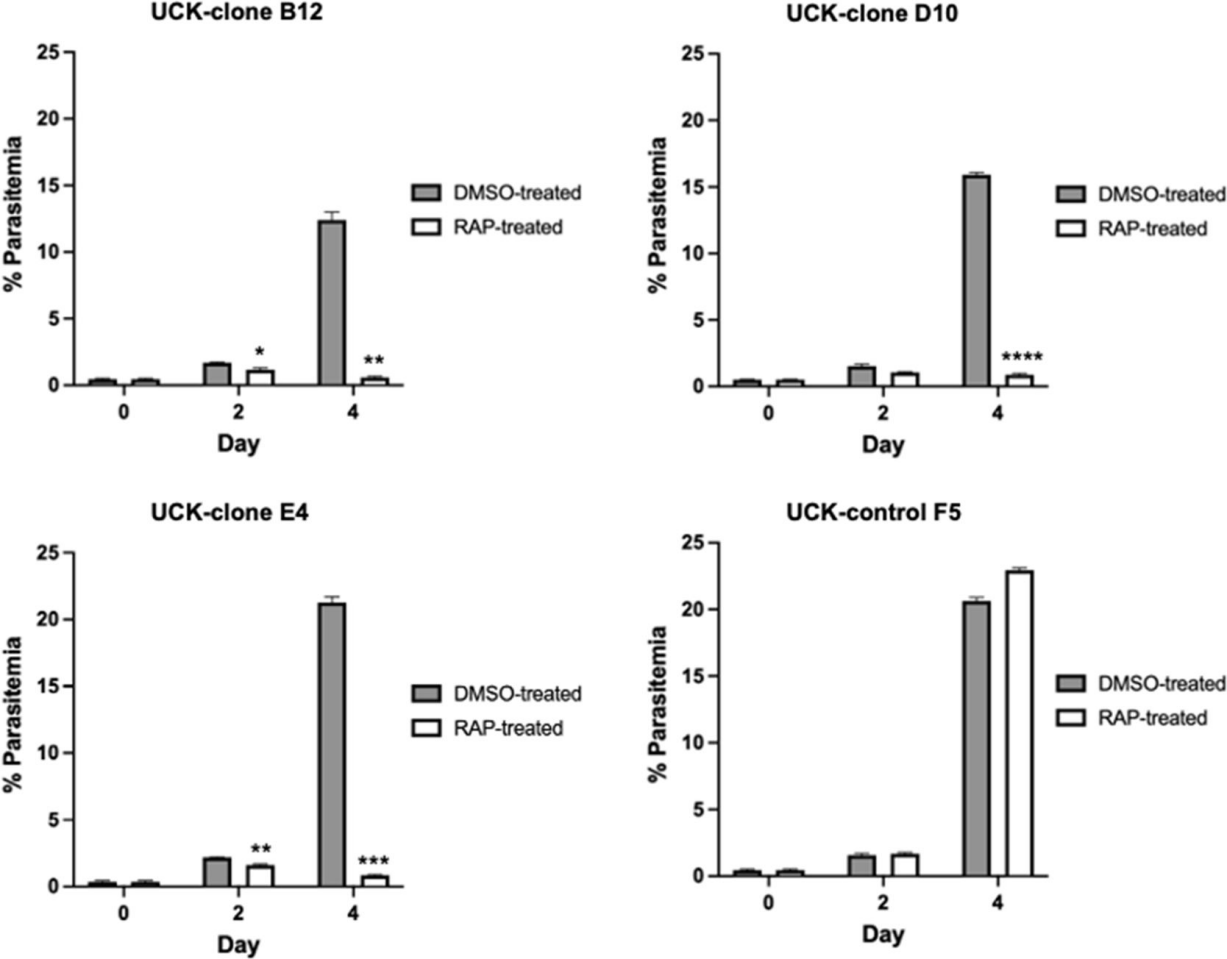
Effect of induced *PfUCK* disruption on parasite growth. Parasitemia values were determined by FACS (each data point represents the mean ± standard error across triplicate counts (100,000 events). Statistical analysis of parasitemia in the RAP-treated parasite culture relative to the control (DMSO treated) culture was conducted using two-way ANOVA (Bonferroni’s multiple comparison test) with ****, *P* < 0.0001; ***, *P* < 0.001; **, *P* < 0.005; *, *P* < 0.05.

To assess the growth effects of disruption of *PfUCK*, a growth assay based on FACS of SYBR green-labeled transgenic parasites was performed over two cycles of intraerythrocytic development for parasites treated with DMSO (control) compared with RAP. Four days following RAP treatment, the parasitemia levels for *UCK-*deleted parasites were significantly less than the controls (Fig. 5). Decreased growth was slightly observable after the first cycle of replication following RAP treatment (i.e., clones B12 and E4). In contrast, the growth of the parasite clone carrying a single *loxP* site was unaffected by RAP treatment. For parasite generations, cycle 1 (days 0–2) and cycle 2 (days 3 and 4), parasitemia levels of UCK-clones B12, D10, and E4 did not increase, indicating that *UCK* is essential for growth and replication of intraerythrocytic parasites.

### Stage-dependent effects of UCK deletion

**Fig 6:**
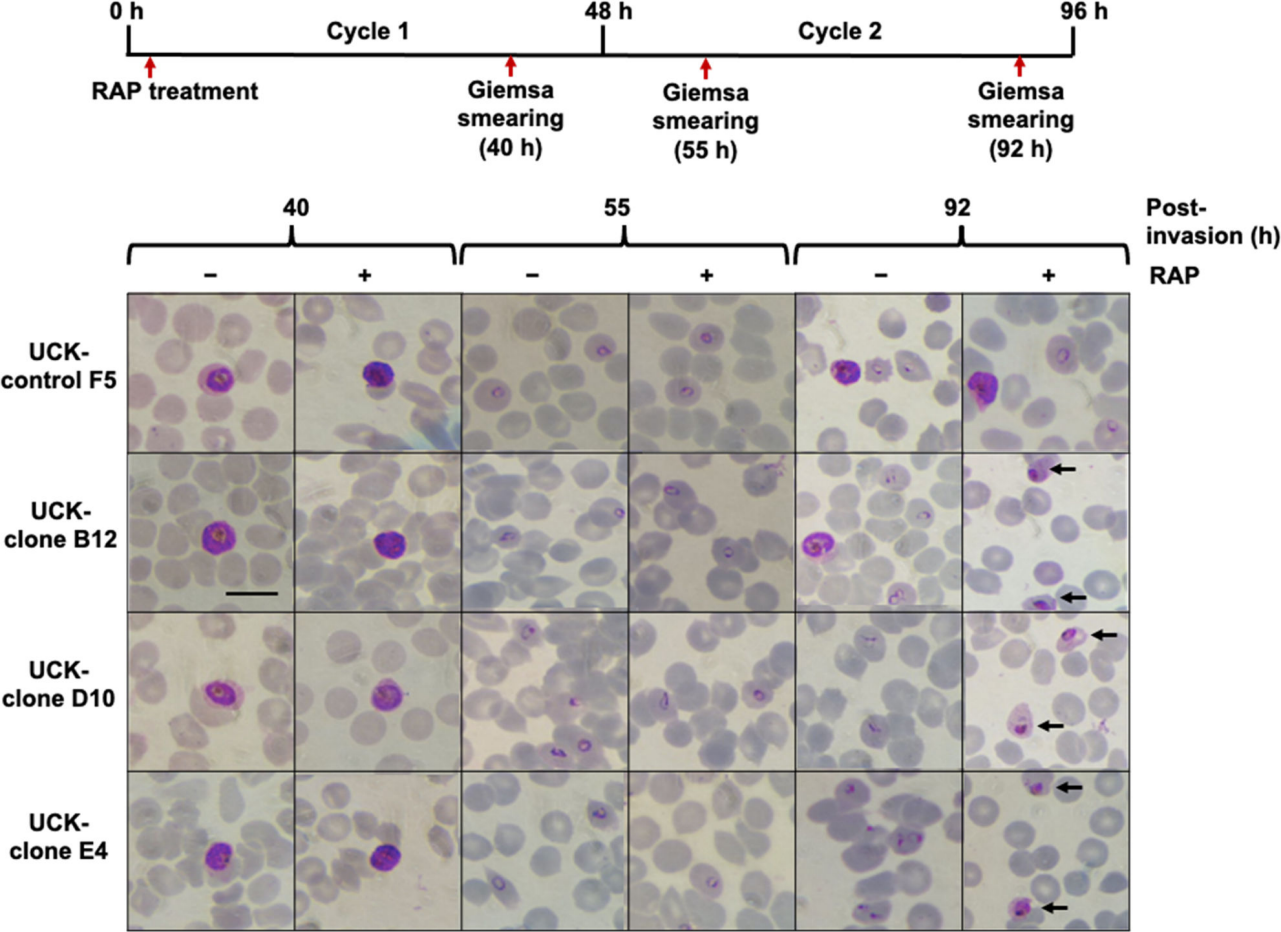
Effect of *PfUCK* truncation on parasite development based on microscopic examination. Giemsa-stained thin blood smearing shows the morphology of the truncated compared to the control (DMSO treated) parasites. Unhealthy trophozoites of disrupted parasite UCK-clones B12, D10, and E4 (RAP treated) were detected in the following cycle. Unhealthy with shrunken trophozoites is indicated by arrows. The time course of sampling of the cultures is shown on top. Scale bar equals 10 µm.

To assess the effects of *UCK* deletion on parasite development, synchronized ring-stage parasites of transgenic clones UCK-clone B12, UCK-clone D10, and UCK-clone E4 were treated with 50 nM RAP (or placebo 0.1% DMSO) for 24 hours and monitored by microscopy. The UCK-control F5 was used as the control for unexcised parasites. Parasite development was monitored using Giemsa-stained smears in comparison to both DMSO-treated parasites and UCK-control F5 control parasites at 40, 55, and 92 hours post-invasion (Fig. 6). The first cycle progressed normally with parasite development from rings to schizonts appearing normal at 40 hours post-invasion and transgenic parasites cycling to new ring-stage parasites at 55 hours. After the next cycle (cycle 2 at 92 hours post-invasion), progression of development had ceased for *UCK-*truncated parasites (UCK-clones B12, D10, and E4 RAP-treated parasites), and they were stalled at the trophozoite stage and appeared unhealthy with shrunken trophozoites. The DMSO-treated control cultures and parasite UCK-control F5 progressed normally with healthy, normal late schizonts and early ring-stage parasites visible. These results support that *UCK* ablation causes a severe defect in parasite development that becomes apparent after the first growth cycle. Hence, *UCK* is essential for growth and replication of *P. falciparum*.

### Biochemical properties of PfUCK

Recombinant PfUCK was expressed and purified for biochemical analyses. To prevent interference from predicted transmembrane domains, the construct had the amino-terminal 23 amino acids truncated. Therefore, the recombinant enzyme PfUCK-Δ23 was utilized in all biochemical analyses.

**Fig 7:**
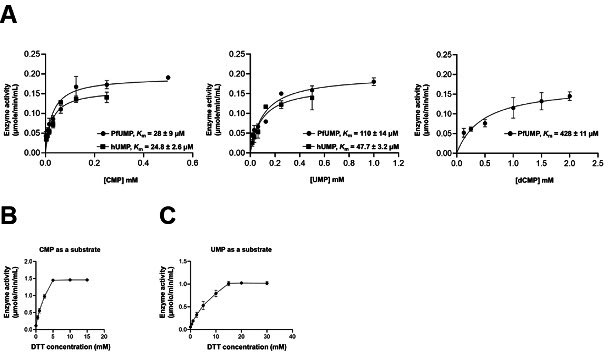
Substrate affinity and reducing dependence of PfUCK. Enzyme activities were assessed by varying substrate concentrations, and *K*_*m*_ values were determined using the Michaelis-Menten equation in GraphPad Prism software (*v* = *V*_max_[*S*]/*K*_*m*_ + [*S*]), where *v* represents the reaction rate, [*S*] denotes the substrate concentration, and *K*_*m*_ signifies the Michaelis constant. Graphs depicting *K*_*m*_ determination for PfUCK and hUCK (**A**) are shown. Additionally, purified protein under a nonreducing condition was tested for its activity by varying DTT concentrations, using CMP (**B**) and UMP (**C**) as substrates.

The kinetic parameters of PfUCK were compared with its human ortholog, hUCK (66). The summarized results are shown in Table 2, and the kinetic plots are presented in Fig. 7A. Enzyme activity assays, utilizing ATP as a phosphate donor, demonstrated PfUCK’s preference for ribonucleoside monophosphates CMP and UMP as substrates over dCMP. PfUCK exhibited the lowest *K*_*m*_ for CMP (28 µM), while UMP displayed a 3.9-fold higher *K*_*m*_ (110 µM). In contrast, the *K*_*m*_ for dCMP was notably higher at 428 µM compared to CMP and UMP. Similar to that of PfUCK, hUCK displayed a *K*_*m*_ for CMP (24.8 µM), while its *K*_*m*_ for UMP was 2.3-fold greater than PfUCK.

**TABLE 2:** PfUCK kinetic parameters

Phosphate acceptor	PfUCK	hUCK	hUCK^*a*^
	*K*_*m*_ (µM)	*K*_*m*_ (µM)	*K*_*m*_ (µM)
UMP	110 ± 14	47.7 ± 3.2	45 ± 10
CMP	28 ± 9	24.8 ± 2.6	20 ± 5
dCMP	428 ± 11	nd	900 ± 100

^*a*^ (66).

The substrate binding affinity of hUCK observed in this study is similar to those previously reported by Pasti et al. (66). Regarding substrate preferences, this investigation revealed that ribonucleoside monophosphates (CMP and UMP) are favored substrates for both human and *Plasmodium* parasites, compared to dCMP. The most preferred substrate for both hUCK and PfUCK was CMP.

We investigated the effect of reducing agents on PfUCK activity, illustrated in Fig. 7B and C, as it was previously reported that reducing agents activate UCK kinase activity (67). As found with hUCK, PfUCK activity was enhanced in response to reducing conditions.

### Inhibitor development for PfUCK

A set of compounds was selected to inhibit UCK activity based on *in silico* screening. Currently, there are no known UCK inhibitors. A homology model of PfUCK was generated using SWISS-MODEL (68), with the UMP/CMP kinase structure from *Dictyostelium discoideum* (PDB ID: 2UKD) as the template. The strategy for the inhibitor design was to identify covalent inhibitors of the enzyme (targeted toward Cys139). Virtual screening was performed using the CovDock tool within Glide (69) focused on modulation of Cys139. Covalent virtual libraries were selected from a number of vendors to include a range of electrophilic warheads thought suitable for cysteine modification, including acrylamide, chloroacetamide, and benzoyl acetonitrile. Screening results for acrylamide and chloroacetamides against PfUCK and human UCK (hUCK) are summarized in Table 3. Among these compounds, the pyrimidin-4-one scaffold was present within three hits accommodated both within chloroacetamide and acrylamide warheads and exhibited significant inhibition activity against PfUCK, with a *K*_*i*_ value in the single micromolar range. Unfortunately, C43 and C21 also inhibited hUCK activity, with a similar *K*_*i*_ value in the same range. Hence, further refinement and selectivity optimization were needed for UCK inhibitors to avoid possible cytotoxicity.

**TABLE 3:** Screening acrylamide and chloroacetamide compounds against PfUCK and hUCK

Compound	Structure	*K*_*i*_—PfUCK (µM)	*K*_*i*_—hUCK (µM)	Selectivity index
C51 (acrylamide)	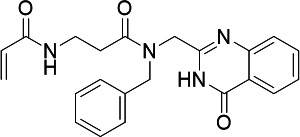	Inactive	nd	nd
C54 (acrylamide)	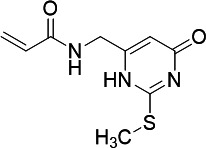	Inactive	nd	nd
C59 (acrylamide)	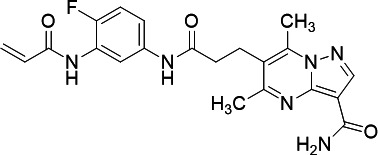	Inactive	nd	nd
C43 (acrylamide/pyrimidin-4-one)	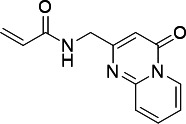	4.15 ± 0.26	4.55 ± 0.52	1.09
C58 (pyrimidin-4-one)	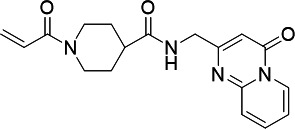	2.11 ± 0.21	3.20 ± 0.38	1.52
C21 (pyrimidin-4-one)	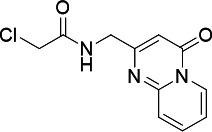	13.72 ± 3.18	2.98 ± 0.50	0.22

Among the benzoylacetonitrile derivatives (Table 4), several (six compounds; C38, C83, C39, C7, C12, and C69) exhibited micromolar level inhibition of PfUCK highlighting C38, which contains a fluoro-group at the meta-position that increases the reactivity resulting in the most potent inhibitor with a *K*_*i*_ value of 1.66 ± 0.27 µM. Based on the structure-activity relationship, the lack of inhibition by C69 is inexplicable. C38, C39 (ortho-chloro derivative), and C7 (meta-chloro derivative) exhibited selectivity for PfUCK with little or no inhibition of hUCK. These results highlight the crucial role of specific substituents in determining both inhibitory potency and selectivity against PfUCK.

**TABLE 4:** Screening benzoylacetonitrile derivative compounds against PfUCK and hUCK

	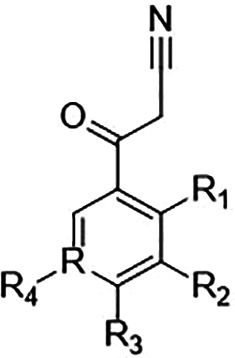							
Compound	Substituent (R)					*K*_*i*_—PfUCK (µM)	*K*_*i*_—hUCK (µM)	Selectivity index
	R	R1	R2	R3	R4			
C38	C	H	–F	H	H	1.66 ± 0.27	No inhibition at 0.5 mM	>600
C69	C	H	–CF3	H	H	No inhibition at 1 mM	nd	
C83	C	H	–CF3	H	–CF3	4.66 ± 0.29	2.79 ± 0.42	0.6
C11	C	H	H	H	H	No inhibition at 1 mM	nd	
C39	C	–Cl	H	H	H	3.64 ± 0.59	No inhibition at 1 mM	>275
C7	C	H	–Cl	H	H	4.26 ± 0.55	No inhibition at 1 mM	>235
C10	C	H	H	–Cl	H	No inhibition at 1 mM	nd	
C64	C	–Cl	–Cl	H	H	No inhibition at 1 mM	nd	
C12	C	H	–Cl	–Cl	H	11.26 ± 2.22	No inhibition at 1 mM	>89
C62	C	H	–Cl	H	–Cl	No inhibition at 1 mM	nd	
C66	C	H	–CH3	H	H	No inhibition at 1 mM	nd	
C80	C	H	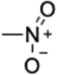	H	H	No inhibition at 1 mM	nd	
C79	C	H	H	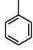	H	No inhibition at 1 mM	nd	
C77	C	H	H	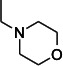	H	No inhibition at 1 mM	nd	
C9	C	H	H	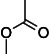	H	insoluble	nd	
C65	C	H	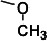	H	H	no inhibition at 1 mM	nd	
C8	C	H	H	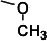	H	no inhibition at 1 mM	nd	
C37	C	H	–Br	H	H	no inhibition at 1 mM	nd	
C67	N	H	–Br	H	H	4.38 ± 1.45	5.31 ± 0.61	1.2

Among the compounds tested, those that inhibited PfUCK were evaluated for their effect on *P. falciparum* growth (IC_50_) (Table 5). The only inhibitor with significant antiparasitic activity was the pyrimidin-4-one derivative C21 with an IC_50_ value of 15.64 ± 1.13 µM (Table 3 includes acrylamide- and pyrimidin-4-one-tested compounds). Regarding the benzoylacetonitrile derivatives, four (C7, C38, C66, and C83) displayed weak inhibitory activities against *P. falciparum* with IC_50_ values exceeding 310 µM (Table S7).

**TABLE 5:** Antiparasitic activities of PfUCK inhibitors

	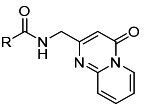	
Compound	Structure	IC_50_ - *—P. falciparum* (3D7)
**C43**		> 1 mM
**C58**		> 1 mM
**C21**		15.64 ± 1.13 µM

## DISCUSSION

This project aimed to test the ability of *in silico* metabolic modeling of *P. falciparum* to identify a novel essential and druggable target and characterize the asserted target PfUCK for antimalarial drug development. The model presented here was developed through the merging of three previously published models (14, 15, 27). In contrast to the Plata et al. (27) and Huthmacher et al. (14) models, where gene expression data were used as additional constraints to model reaction fluxes, transcriptomic data were not included in our model as there are a number of factors that can result in delays from gene expression and the corresponding metabolic reaction (70, 71). Instead, to improve the accuracy of model predictions, we have incorporated experimentally derived metabolomics data, which hasve been shown to contribute to the accuracy of model prediction since metabolomics provide better insight oninto metabolic reactions (72). Through flux-balance analysis, our model identified a set of gene-associated proteins that were predicted to be essential, and hence, potential drug targets and the top-ranked *PfUCK* demonstrated validation of the model as an essential gene.

*PfUCK* is essential for growth with parasites containing the knockout of *UCK* arrested in the trophozoite stages of asexual development. Although the generation of the inducible mutants is a laborious multistep process, it definitively demonstrated the necessity of *PfUCK,* in contrast to a subtractive process of classification of genes (i.e., genes that cannot be mutated) that may generate false-positive essential genes. The distinction between whether disruption is growth- limiting or lethal is less clear. The timing of arrest in the life cycle was expected, given the demand for pyrimidines during the stages of DNA and RNA synthesis when *PfUCK* is most highly expressed. High expression of *UCK* is detected in the early ring stage before declining in the mid-ring and late-ring parasites (73). The halt in progression through the life cycle was delayed in parasites with the *UCK* ablation. Dysmorphic trophozoites were not observed until two cycles following induction of the gene excision. This may be due to delays during the gene excision process in the rate of recombination. Prior research on the generation of conditional gene knockouts in *P. falciparum* using the DiCre recombinase system found that timing of excision can be impacted by drug concentration, timing of drug addition in life cycle, and duration of exposure (19). Excision was detected significantly after 20 hours of treatment and required >30 hours to achieve maximum excision levels. Alternatively, the half-life of *PfUCK* mRNA and the enzyme may permit sufficient amounts of uridine/cytidine phosphorylation for a single cycle of replication before halting developmental progression.

There are needs for the development of both slower-acting and rapid antimalarial agents (74). The delayed response to disruption may indicate PfUCK will be an effective target for slower-acting antimalarial agents. Slower action is better used for preventing recrudescence and for longer-term protection. Recommendations are for combinations of fast-acting and slower-acting drugs, as combination therapy is essential due to the rapid development of resistance. Pyrimidine synthesis and mitochondrial cytochrome *bc*_1_ are valuable targets for liver stages of parasite development as well as asexual blood stages (75). PfUCK inhibition is well placed to be combined with drugs targeting these pathways. For example, dihydroorotate dehydrogenase inhibitors or the cytochrome *bc*_1_ inhibitor atovaquone (8, 9).

Disulfide bonds appear crucial to PfUCK activity based on its sensitivity to reducing agents. This may indicate the importance of the conserved cysteine residues in PfUCK (Cys298 and Cys307) that modelling suggests haves the potential to form disulfide bonds. These cysteines may be vulnerable to irreversible inhibitors, analogous to irreversible kinase inhibitors for cancer, that form covalent bonds with cysteine (76). Irreversible inhibitors have the potential to be long acting due to the residence time, possibly less frequent dosing due to low turnover, and have less resistance development binding to essential cysteine residues. Additionally, the oxidant susceptibility may highlight how valuable UCK can prove as a target because of the high sensitivity of the parasite to redox balance and oxidant stress.

The sensitivity of PfUCK to micromolar-level range inhibitors with one exhibiting antiparasitic activity (IC_50_ 15.64 ± 1.13 µM) is promising for antimalarial drug development. Our modeling predicted interaction of inhibitors with Cys139. Future studies will assess the covalent attachment of the inhibitors using kinetic assays and mass spectrometry, as the acrylamide warhead compounds have the potential for cysteine interaction (77). This work would also benefit from co-crystals of PfUCK with inhibitors to facilitate structure-based drug design.

Based on the findings of the studies herein, the assertion of gene essentiality in a GSM based on experimental biomass and experimental flux measurements was founded in an inducible gene knockout, demonstrating that UCK is essential in *Plasmodium*. Further development of inhibitors of this essential enzyme with its redox sensitivity and potential for irreversible inhibition may provide a valuable avenue to antimalarial development.

## SUPPLEMENTAL MATERIAL

Data S1 aac.00459-25-s0001.txt The model coding.

Supplemental material aac.00459-25-s0002.docx Fig. S1; Tables S1, S5, S7; Data S2.

Supplemental tables aac.00459-25-s0003.xlsx Tables S2 to S4, S6.

ASM does not own the copyrights to Supplemental Material that may be linked to, or accessed through, an article. The authors have granted ASM a non-exclusive, world-wide license to publish the Supplemental Material files. Please contact the corresponding author directly for reuse.
