# Supplementary material for: Validated antimalarial drug target discovery using genome-scale metabolic modeling
Source: Antimicrob Agents Chemother. 2025 Sep 26;69(11):e00459-25. doi: 10.1128/aac.00459-25 (PMC12588083; doi:10.1128/aac.00459-25)
Supplement: Supplemental material — Fig. S1; Tables S1, S5, S7; Data S2. [file aac.00459-25-s0002.docx]

**Supplementary Figure 1. Summary of predicted essential genes.** Single gene knockouts with COBRApy (1) were simulated using the schizont stage metabolic model to predict essential genes. The graph shows a comparison of the predicted essential genes and their associated reactions with experimentally validated essential reactions. The proportions of true positive predictions (overall, lethal and growth limiting knockouts) were compared against the proportion of genes in the model that are in the gold standard list and the corresponding enrichment hypergeometric p-values were calculated.

Supplementary Table 1 HPLC program for the separation of amino acids. Percentage composition and flow rates of the mobile phase at given times as part of the ramp gradient reverse-phase chromatography for the separation of amino acids in spend media.

| **Time (min)** | **Percent Eluent A** | **Percent Eluent B** | **Flow rate (ml/min)** |
| --- | --- | --- | --- |
| 0.0 | 98% | 2% | 0.200 |
| 60.0 | 55% | 45% | 0.200 |
| 60.5 | 98% | 2% | 0.200 |
| 70.5 | 98% | 2% | 0.200 |

**Supplementary Tables 2 to 4. List of experimentally validated essential genes from literature search, PlasmoGEM, and *piggyBac* saturation mutagenesis.** Model-predicted essential genes were compared against the list of experimentally validated genes obtained from literature search and PlasmoGEM (2). Gene essentiality as determined by *piggyBac* saturation mutagenesis (3) were incorporated into Supplementary Tables 2 and 3 for comparison.

Please see attached Excel file “Additional Supplementary Tables.xlsx”

**Supplementary Table 5 Experimental glucose and amino acid flux measurements.** Spent media from synchronised *Plasmodium falciparum* 3D7 cultures were collected at 6, 18, 30, 36, 42 and 48 hours post synchronisation (hps), and glucose and amino acid flux (in mmol/gDW/hr ± SD) were calculated points to represent the early to mid-ring stage, late trophozoite stage and late schizont stage, respectively. A positive flux indicates movement of the metabolite into the infected red blood cell while a negative flux indicates efflux of the metabolite into the media.

| **Metabolite** | **6 - 18 hps** | **30 - 36 hps** | **42 - 48 hps** |
| --- | --- | --- | --- |
| D-Glucose | 1.3 ± 0.82 | 3.2 ± 1.2 | 0.60 ± 0.21 |
| L-Alanine | -0.023 ± 0.017 | -0.041 ± 0.036 | 0.00 ± 0.024 |
| L-Arginine | 0.072 ± 0.11 | -0.15 ± 0.49 | 0.22 ± 0.35 |
| L-Asparagine | -0.0094 ± 0.011 | -0.10 ± 0.12 | 0.070 ± 0.17 |
| L-Aspartic acid | 0.0048 ± 0.011 | 0.00 ± 0.029 | 0.019 ± 0.058 |
| L-Glutamic acid | -0.019 ± 0.0081 | -0.056 ± 0.024 | 0.0047 ± 0.077 |
| L-Glutamine | 0.00 ± 0.023 | -0.40 ± 0.63 | 0.36 ± 0.72 |
| Glycine | -0.014 ± 0.028 | -0.0093 ± 0.12 | 0.037 ± 0.17 |
| L-Histidine | -0.0073 ± 0.013 | -0.066 ± 0.095 | 0.066 ± 0.076 |
| L-Isoleucine | 0.017 ± 0.023 | -0.062 ± 0.14 | 0.081 ± 0.14 |
| L-Leucine | 0.28 ± 0.065 | -0.14 ± 0.070 | 0.047 ± 0.10 |
| L-Lysine | 0.00 ± 0.064 | 0.038 ± 0.20 | 0.073 ± 0.29 |
| L-Methionine | 0.0024 ± 0.0082 | -0.028 ± 0.025 | 0.028 ± 0.025 |
| L-Phenylalanine | -0.0099 ± 0.016 | -0.035 ± 0.0086 | 0.025 ± 0.023 |
| L-Serine | -0.019 ± 0.021 | -0.062 ± 0.091 | 0.062 ± 0.11 |
| L-Threonine | -0.014 ± 0.026 | -0.038 ± 0.050 | 0.028 ± 0.099 |
| L-Tryptophan | 0.0026 ± 0.0045 | 0.010 ± 0.036 | 0.010 ± 0.024 |
| L-Tyrosine | -0.0051 ± 0.0044 | -0.031 ± 0.031 | -0.046 ± 0.17 |
| L-Valine | -0.036 ± 0.098 | -0.0096 ± 0.022 | 0.024 ± 0.12 |

**Supplementary Table 6. List of genes in the iFT342 model.** All 342 genes in the iFT342 genome scale metabolic model with their corresponding product description, and gene essentiality based on GSM model prediction, as well as experimental validation from literature and PlasmoGEM (2).

Please see attached Excel file “Additional Supplementary Tables.xlsx”

Supplementary Table 7 Antiparasitic activities of benzoylacetonitrile derivative compounds

**
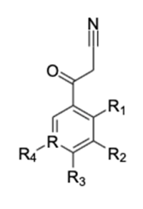
**

| **Compound** | **Substituent (R)** | | | | | **IC_50_ - *P. falciparum* (3D7), µM** |
| --- | --- | --- | --- | --- | --- | --- |
|  | **R** | **R1** | **R2** | **R3** | **R4** |  |
| C38 | C | H | –F | H | H | 338.56 ± 26.39 |
| C69 | C | H | –CF3 | H | H | nd |
| C83 | C | H | –CF3 | H | –CF3 | 316.63 ± 50.77 |
| C11 | C | H | H | H | H | nd |
| C39 | C | –Cl | H | H | H | no inhibition at 1 mM |
| C7 | C | H | –Cl | H | H | 379.98 ± 50.09 |
| C10 | C | H | H | –Cl | H | nd |
| C64 | C | –Cl | –Cl | H | H | nd |
| C12 | C | H | –Cl | –Cl | H | no inhibition at 0.5 mM |
| C62 | C | H | –Cl | H | –Cl | nd |
| C66 | C | H | –CH3 | H | H | 309.93 ± 57.87 |
| C80 | C | H | 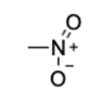 | H | H | nd |
| C79 | C | H | H | 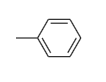 | H | nd |
| C77 | C | H | H | 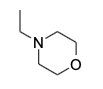 | H | nd |
| C9 | C | H | H | 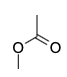 | H | nd |
| C65 | C | H | 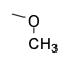 | H | H | nd |
| C8 | C | H | H | 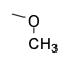 | H | nd |
| C37 | C | H | –Br | H | H | nd |
| C67 | N | H | –Br | H | H | 531.15 ± 61.18 |

**Supplementary**

**Data S2: Plasmid construction for disrupting the *UCK* gene**

To validate *UCK* as a drug target, a CRISPR/Cas9-based strategy combined with DiCre-mediated recombination was employed. The repair plasmid was designed to disrupt the UCK gene by inserting one loxP site upstream within intron 2 and a second loxP site downstream at the end of the coding sequence. This design enables RAP-inducible excision of the majority of the active site, resulting in a conditional knockout for functional analysis. The UCK locus was then modified using CRISPR/Cas9 by co-transfecting the plasmid repair and pUF1-Cas9 into the DiCre-expressing P. falciparum B11 line (4). After successful integration, transgenic parasites were treated with RAP to induce the excision of the UCK gene. Parasite growth and developmental progression were subsequently monitored to evaluate *UCK* function.


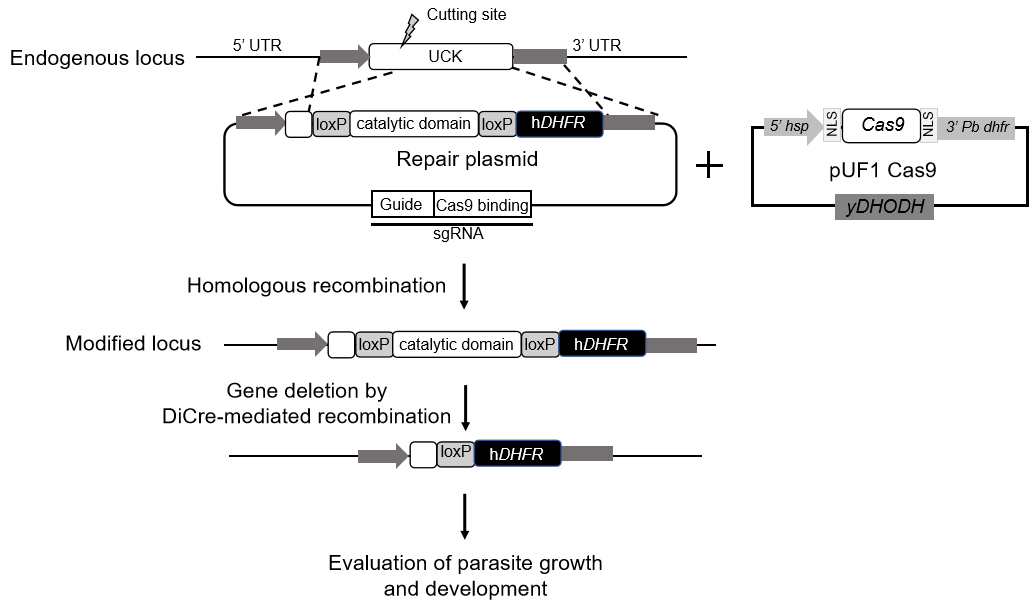


Two repair plasmids, namely pL6-UCK_loxP-sgRNA4-native E3-I3 and pL6-UCK_loxP-sgRNA4-native E3-ΔI3 were constructed in this study. Both plasmids were derived from the pL6-eGFP plasmid (5). The plasmid pL6-UCK_loxP-sgRNA4-native E3-I3 retains both exon 3 and intron 3 in their native sequence, while pL6-UCK_loxP-sgRNA4-native E3-ΔI3 was generated by removing the native intron 3 sequence from the former construct.


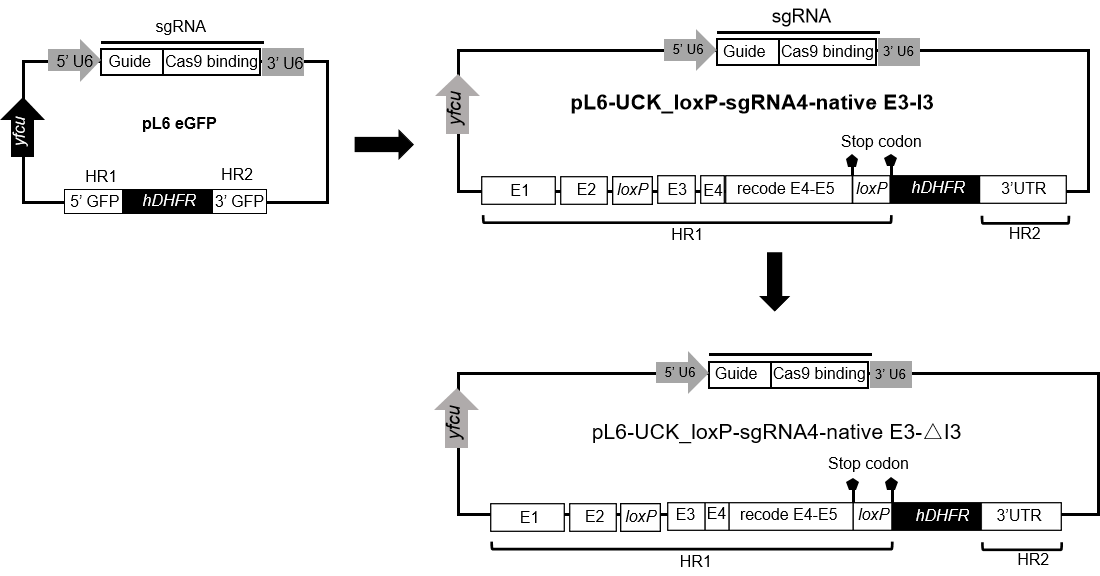


The first plasmid, named pL6-UCK_loxP-sgRNA4-native E3-I3, was developed based on the pL6-eGFP backbone. This repair plasmid includes both a single guide RNA (sgRNA) and a donor DNA template required for homology-directed repair, in which the hDHFR gene is flanked by two homology regions (HR1 and HR2). To generate the construction, the GFP sequences (GFP 5′ and GFP 3′) in the original pL6-eGFP plasmid were replaced with homology arms specific to the gene of interest (GOI). The sgRNA targeting sequence was then inserted into the BtgZI site as described by Global et al. (5).

In the initial step, sgRNA4 (CATAATGAATTAAATGACCA), identified using the sgRNA design tool in Benchling (6), was introduced into the pL6-eGFP plasmid using the NEBuilder HiFi DNA Assembly Kit (New England BioLabs Inc., USA). Subsequently, the donor DNA sequence containing the homology-directed repair 1 (HDR1) region—corresponding to nucleotides 113–1108 of the genomic DNA—was inserted. In this arrangement, the upstream loxP sequence was positioned within intron 2 (between nucleotide 684 and 720), followed by the recoded *PfUCK* gene (nucleotides 1109-1765), the downstream loxP site, the *hDHFR* gene serving as the selectable marker, and the 3’ UTR sequence forming the homology-directed repair 2 (HDR2). This process was facilitated using the NEBuilder HiFi DNA assembly kit (NEB Inc, USA) to integrate these elements into the plasmid pL6 eGFP.

The repair plasmid pL6-UCK_loxP-sgRNA4-native E3-ΔI3 was derived from the plasmid pL6-UCK-sgRNA4-native E3-I3 by eliminating the native intron 3 sequence. To accomplish this, two sets of primers were employed. The HDR1 forward primer (5’-CTTTCCGCGGGGAGGACTAGTACATAAATAGAACTAATGGTT-3’) and the native Exon3_reverse primer (5’-CGTTGTTTATACAATCCTCA-3’) were used to amplify the fragment containing nucleotides 113–864 from the plasmid pL6-UCK-sgRNA4-native E3-I3. Subsequently, the exon4 forward primer (5’-TGAGGATTGTATAAACAACGGTAAAATCGTGCCGG-3’) and the native exon4 reverse primer (5’-TTTTTTTTCAACACCAGATTCACCCTGGTCGTT-3’) were used to amplify the fragment containing nucleotides 1026-1108 from the genomic DNA, with the exon4 forward primer designed to exclude the intron 3 sequence. Consequently, these amplified fragments were inserted into the plasmid pL6-UCK-sgRNA4-native E3-I3, with no alterations made to the recoded *PfUCK* gene (nucleotides 1109-1765), the downstream loxP site, the *hDHFR* gene, or the 3’ UTR sequence, which serves as the homology-directed repair 2 (HDR2).

**References**

1. Ebrahim A, Lerman JA, Palsson BO, Hyduke DR. 2013. COBRApy: COnstraints-Based Reconstruction and Analysis for Python. 1. BMC Syst Biol 7:74.

2. Schwach F, Bushell E, Gomes AR, Anar B, Girling G, Herd C, Rayner JC, Billker O. 2015. PlasmoGEM, a database supporting a community resource for large-scale experimental genetics in malaria parasites. Nucleic Acids Res 43:D1176–D1182.

3. Zhang M, Wang C, Otto TD, Oberstaller J, Liao X, Adapa SR, Udenze K, Bronner IF, Casandra D, Mayho M, Brown J, Li S, Swanson J, Rayner JC, Jiang RHY, Adams JH. 2018. Uncovering the essential genes of the human malaria parasite *Plasmodium falciparum* by saturation mutagenesis. Science 360:7847.

4. Perrin AJ, Collins CR, Russell MRG, Collinson LM, Baker DA, Blackman MJ. 2018. The Actinomyosin Motor Drives Malaria Parasite Red Blood Cell Invasion but Not Egress. mBio 9:10.1128/mbio.00905-18.

5. Ghorbal M, Gorman M, Macpherson CR, Martins RM, Scherf A, Lopez-Rubio J-J. 2014. Genome editing in the human malaria parasite Plasmodium falciparum using the CRISPR-Cas9 system. Nat Biotechnol 32:819–821.

6. https://www.benchling.com/
